# Tissue factor (coagulation factor III): a potential double-edge molecule to be targeted and re-targeted toward cancer

**DOI:** 10.1186/s40364-023-00504-6

**Published:** 2023-06-06

**Authors:** Seyed Esmaeil Ahmadi, Ashkan Shabannezhad, Amir Kahrizi, Armin Akbar, Seyed Mehrab Safdari, Taraneh Hoseinnezhad, Mohammad Zahedi, Soroush Sadeghi, Mahsa Golizadeh Mojarrad, Majid Safa

**Affiliations:** 1 Departments of Hematology and Blood Banking, Faculty of Allied Medicine, Iran University of Medical Sciences, Tehran, Iran; 2 Department of Immunology, School of Medicine, Mazandaran University of Medical Sciences, Sari, Iran; 3 Department of Hematolog, Faculty of Allied Medicine, Bushehr University of Medical Sciences, Bushehr, Iran; 4 Department of Medical Biotechnology, School of Allied Medicine, Iran University of Medical Sciences, Tehran, Iran; 5 Faculty of Science, Engineering and Computing, Kingston University, London, UK; 6 Shahid Beheshti Educational and Medical Center, Kashan University of Medical Sciences, Kashan, Iran

**Keywords:** Tissue factor, Cancer, Metastasis, Angiogenesis, Targeted therapy, Re-Targeted therapy

## Abstract

Tissue factor (TF) is a protein that plays a critical role in blood clotting, but recent research has also shown its involvement in cancer development and progression. Herein, we provide an overview of the structure of TF and its involvement in signaling pathways that promote cancer cell proliferation and survival, such as the PI3K/AKT and MAPK pathways. TF overexpression is associated with increased tumor aggressiveness and poor prognosis in various cancers. The review also explores TF's role in promoting cancer cell metastasis, angiogenesis, and venous thromboembolism (VTE). Of note, various TF-targeted therapies, including monoclonal antibodies, small molecule inhibitors, and immunotherapies have been developed, and preclinical and clinical studies demonstrating the efficacy of these therapies in various cancer types are now being evaluated. The potential for re-targeting TF toward cancer cells using TF-conjugated nanoparticles, which have shown promising results in preclinical studies is another intriguing approach in the path of cancer treatment. Although there are still many challenges, TF could possibly be a potential molecule to be used for further cancer therapy as some TF-targeted therapies like Seagen and Genmab’s tisotumab vedotin have gained FDA approval for treatment of cervical cancer. Overall, based on the overviewed studies, this review article provides an in-depth overview of the crucial role that TF plays in cancer development and progression, and emphasizes the potential of TF-targeted and re-targeted therapies as potential approaches for the treatment of cancer.

## Introduction

Millions of people worldwide suffer from cancer, a disease with a variety of factors contributing to its development and progression. Tissue factor (TF) is one of the factors that has emerged in recent years as being important in cancer [1–3]. TF is a transmembrane glycoprotein known as thromboplastin that is present on the surface of many different types of cells [4–6], including cancer cells [2, 3, 7, 8]. TF plays a critical role in the coagulation cascade, the process by which blood clots form in order to stop bleeding [9–12]. A complex sequence of reactions occurs when TF comes into contact with blood and ultimately leads to the formation of a clot [13]. In normal circumstances, this process is tightly regulated and occurs only when necessary to prevent excessive bleeding [14]. However, TF can be dysregulated in cancer, leading to tumor growth and spread [15]. TF contributes to cancer by promoting angiogenesis, which is the process of forming new blood vessels that carry nutrients and oxygen to tumors. A key factor in angiogenesis is the vascular endothelial growth factor (VEGF), which can be stimulated by TF. In this way, the tumor is nourished and can grow and spread as a result of the formation of blood vessels [16]. Cancer cells can also spread from the primary tumor to other parts of the body through metastasis, which is caused by TF. It stimulates the release of matrix metalloproteinases (MMPs), which dissolve the extracellular matrix (ECM) that surrounds the cells [17]. It is possible for cancer cells to invade surrounding tissues and spread to distant parts of the tumor origin as a result of this. Furthermore, TF contributes to cancer by promoting inflammation [18]. Signaling molecules such as cytokines are stimulated by TF, which regulate immune and inflammatory responses and several types of cancer are known to be affected by chronic inflammation [8, 19].

TF has been found to be up-regulated in a variety of different types of cancer, including breast, lung, colon, and pancreatic cancer [8]. These findings suggest that TF may be a promising target for cancer therapy [15]. TF is currently being targeted in cancer using different approaches, including antibodies, inhibitors, and nanoparticles [20]. Additionally, re-targeting TF shows promise as a cancer therapy. Re-targeting TF works by redirecting its pro-coagulant effects toward tumor blood vessels while sparing normal tissues [21]. A method of achieving this is to conjugate TF-targeting agents to clotting factor VIIa (FVIIa), which binds TF and initiates the coagulation cascade. When TF-FVIIa complexes are formed, they can activate coagulation selectively in the tumor microenvironment, inhibiting tumor growth and disrupting vessels [20]. Preclinical studies have tested several re-targeting strategies for TF-FVIIa, including antibodies, peptides, and nanoparticles. It has been demonstrated that an antibody–drug conjugate (ADC) targeting TF-expressing tumor cells, called ICON-1, selectively kills TF-expressing tumor cells in vitro and inhibits tumor growth in vivo [13, 22–26]. A second approach is to use TF-targeted nanoparticles to deliver anti-cancer drugs or imaging agents to TF-expressing tumors. Cancer therapy based on TF re-targeting has great potential, but more studies are needed to evaluate its safety and effectiveness in clinical trials. Other diseases, such as sepsis and thrombosis, where TF can be dysregulated, may benefit from TF re-targeting approaches [13, 22–26].

Recent studies suggest that TF is a useful surface target in 50–85% of patients with triple-negative breast cancer [27]. Another one suggests that TF isoforms and downstream signaling are promising novel therapeutic targets in malignant diseases [1]. Also, there is a strong relationship between TF and cancer and that many cancer cells express high levels of both full-length TF (flTF) and alternatively spliced TF (asTF) [16]. A key role of TF in cancer is that it promotes angiogenesis, metastasis, and inflammation. By understanding how TF plays a role in cancer, new therapies can be developed that target this molecule and potentially improve outcomes for patients with cancer. The complex interactions between TF and cancer require much more research, but targeting this molecule might lead to new and effective cancer treatments. It's worth noting that a variety of therapies have been created to target TF, such as monoclonal antibodies, small molecule inhibitors, and immunotherapies. These treatments have been shown to be effective in various types of cancer in both preclinical and clinical studies. Another interesting approach in cancer treatment is the use of TF-conjugated nanoparticles to re-target TF towards cancer cells, which has shown promising results in preclinical studies. Although there are still many challenges, TF may have potential as a molecule for further cancer therapy. Some TF-targeted therapies, such as Seagen and Genmab's tisotumab vedotin, have already received FDA approval for cervical cancer treatment. Overall, this review article provides a thorough overview of the important role that TF plays in cancer development and progression, and highlights the potential of TF-targeted and re-targeted therapies as promising approaches for cancer treatment.

## TF structure

TF (also known as thromboplastin, coagulation factor III, F3 or (CD142) is a transmembrane glycoprotein receptor for coagulation factors VIIa and X with a molecular weight of 47 kDa is located on the different cells. TF can initiate blood coagulation upon binding to FVIIa [4, 20, 28–30]. TF is a highly conserved factor, so it is expressed in invertebrates such as horseshoe crabs to vertebrates such as humans [31]. There are six exons in the TF gene: Exons 2 to 5 encode the extracellular domain, which binds factors VII and X; exon 1 encodes the N-terminal signal sequence that is eliminated by proteolytic cleavage during the transport of TF (TF) to the plasma membrane; The cytoplasmic and transmembrane domains are encoded by exon 6 [32]. Furthermore, the intracellular region of TF contains two potential phosphorylation sites, which suggests that this protein participates in intracellular activities [33]. According to its primary sequence, TF has structural similarities with the cytokine receptor family II (most notably the IFN-Y receptor). The plasma coagulation protease cascades are started when the TF extracellular domain binds factor Vlla and functions as its catalytic cofactor, generating thrombin and forming fibrin. The coagulation protease factor VIIa attached to the extracellular domain of TF with sub-nanomolar affinity, activating the protease allosterically and effectively cleaving protein substrates, primarily factor X. This protein is considered to have biological activities beyond fibrin coagulation. It is implicated in a variety of cellular functions, including formation of the embryonic vessels [34], embryogenesis, inflammation, cellular signaling, cell migration and, tumor growth. Additionally, the expression of TF in malignancies promotes tumor angiogenesis and hematogenous metastasis [35, 36].

**Fig. 1:**
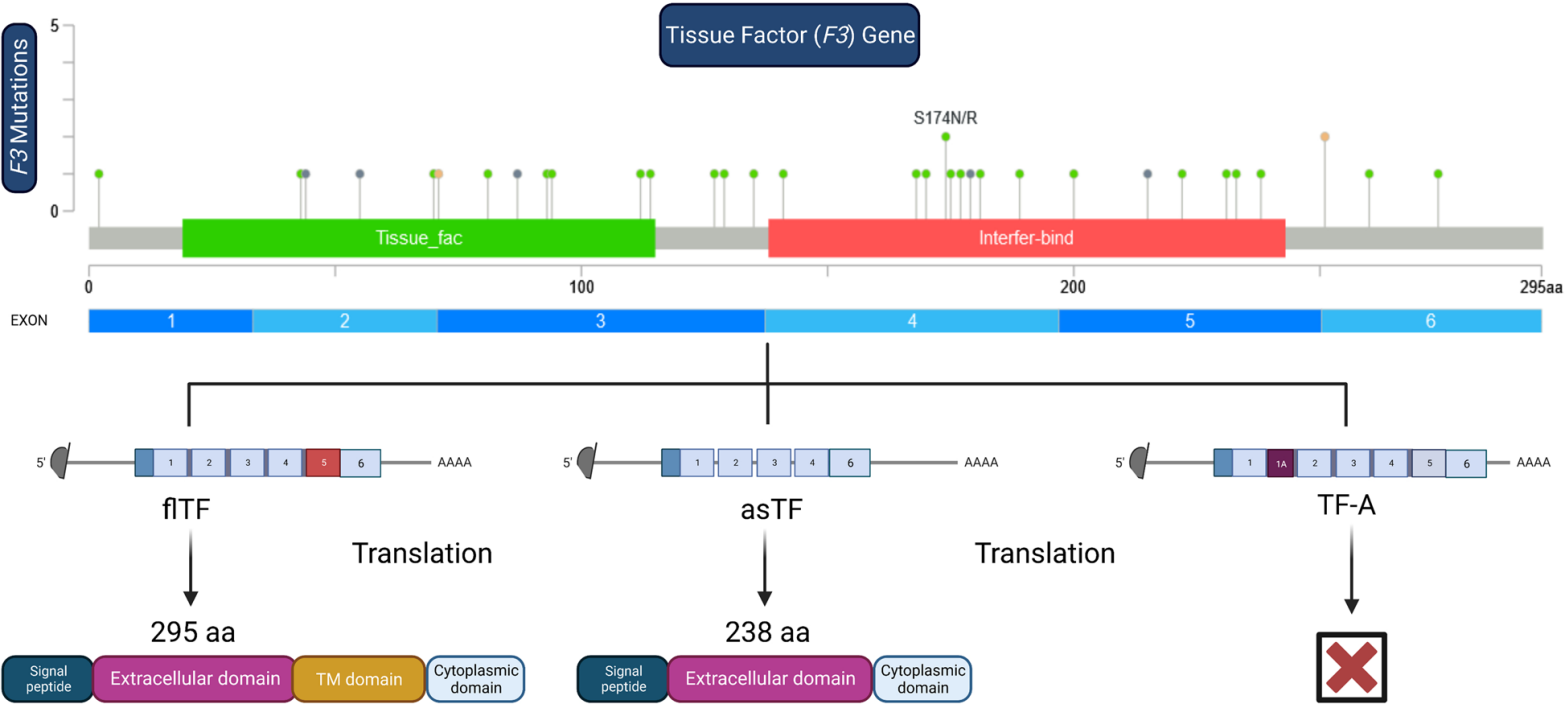
A schematic representation of the mRNA splicing products of *F3* gene is shown. *F3* gene, encodes six exons that form open reading frames; five introns are removed during mRNA processing. Three distinct mRNA products are produced, each of which contains coding sequences. By alternative splicing exon 5 is excluded, which results in the production of TF with a shorter length (238 aa) compared to flTF (295 aa). TF-A is a newly identified transcript of the TF gene that is produced by an alternative splicing mechanism involving the first intron. This process causes the incorporation of a sequence from the first intron known as exon IA into the transcript. Moreover, there is a mutation map of *F3* gene, showcasing the mutation spots (Produced by www.cbioportal.org). TF: tissue factor; aa: amino acid; asTF; alternatively spliced TF; flTF: full-length TF; TF-A: alternative exon 1A-tissue factor; TM domain: Transmembrane domain

TF is found in three forms: alternative exon1A-tissue factor (TF-A), asTF and flTF. Human flTF is a glycoprotein with a total of 295 amino acids (Fig. 1). It is made up of an extracellular domain (residues 1–219), a transmembrane domain (residues 220–242), and an intracellular C-terminal domain (residues 243–263) [37]. The transmembrane domain of asTF is absent, so it is only secreted. Although asTF is not particularly pro-coagulant, it takes part in non-hemostatic functions. As opposed to flTF, asTF can bind integrins without the assistance of FVII [20]. The TF-A variant is a novel transcript of the TF gene that is generated through alternative splicing of the first intron. This process results in the inclusion of an additional sequence derived from an internal sequence of the first intron, referred to as exon IA, and is known as TF-A. Studies have shown that TF-A is preferentially expressed in tumor cells, and its expression level is higher in tumor cells than in normal cells. The upregulation of TF-A in tumor cells may be involved in tumor angiogenesis and metastasis. The high-level expression of the TF-A transcript in tumor cells may have diagnostic and staging utility for various solid tumors. However, further research is needed to fully understand the implications and mechanisms of TF-A upregulation in tumor cells (Fig. 1) [37].

As a result of vascular injury, TF binds to bloodborne FVII or FVIIa with great affinity, triggering the extrinsic pathway to initiate the coagulation cascade. It is composed of a complicated series of proteolytic reactions and a regulatory feedback loop. After that, the zymogen factor (FX) is attached to the binary catalytic complex TF-VIIa, which cleaves to create FXa. FXa separates from the ternary complex and interacts with its cofactor FVa to transform prothrombin into thrombin. Thus, fibrin is formed, platelets are activated, and blood clots are formed [38]. It is commonly assumed that TF is derived from an ancestral gene that also developed the cytokine receptor family. TF appears to be the oldest cytokine classII receptor family member. Some reports highlighted the high degree of similarity in TF structure with the superfamily of interferon receptors (IFNRs) [39]. The region of the TF that is structurally similar to class II cytokine receptors is known as the intracellular C-terminal domain [20].

A difference between asTF and flTF is that flTF adheres to the cell membrane and is involved in signaling and tumor progression through the activation of Par2. Unlike flTF, asTF has little prothrombogenic activity, but is more associated with angiogenesis, survival, and tumor growth [2, 40]. The important point is that, unlike flTF, asTF controls angiogenesis through binding to integrin without connection with PAR [41]. Human tissue factor pathway inhibitor (TFPI), is a regulatory protein in hemostatic system which consists of three Kunitz type domains. These three domains are: N-terminal acidic region followed by the first Kunitz domain (K1), the first linker region which indicates the second Kunitz domain (K2), and the second linker region which indicates the third Kunitz domain (K3). Besides, TFPI has a C-terminal region with basic essence. The K1 domain inhibits the complex of TF-VIIa and K2 domain inhibits factor Xa. Contrary to the K1 and K2 domains, K3 domain has no direct protease inhibiting functions [42]. Noteworthy that its first domain binds to active factor 7 (VIIa), and its second domain binds to active factor 10, thereby inhibiting TF (VIIa) and active factor 10 (Xa) [43].

TF signaling is influenced by phosphorylation and two phosphorylation sites (Ser253 and Ser258, respectively) have been discovered in the C-terminus of TF [20]. A variety of cellular functions, such as gene transcription, protein translation, apoptosis, and cytoskeletal rearrangement, are regulated by TF-dependent signaling, which works together to enable the cell to adapt to its extracellular environment appropriately [38]. The underlying molecular pathways by which cancerous cells communicate with the bloodstream to develop cancer have been identified [44]. Cancer patients usually have an active hemostatic system. A natural physiological function is taken advantage of by cancer cells to promote tumor dissemination. Malignancy progression, cancer-related thrombosis, and metastasis are all attributed to TFs' role in coagulation activation. Nevertheless, it is still unclear how to direct TF signaling pathways affect cancer [28, 45]. Additionally, it has been revealed that the degree of TF expression in different tumor types correlates with their aptitude for metastasis [28]. Up-regulated TF levels are typically observed in a variety of malignancies and also in metastatic cells through integrated oncogenic activity and tumor suppressor inactivation [46]. The TF directs the assembly of VIIa with substrate X to form a ternary complex, which produces product Xa during the coagulation initiation phase. FVIIa and FXa, together, intensely stimulate tumor cell migration by involving Protease-activated receptors (PAR) [29]. Besides initiating blood coagulation, TF is demonstrated to have an essential function in various physiological procedures such as tissue repairing, inflammation, angiogenesis, tumor metastasis, and embryogenesis [28, 29]. A crucial component of these nonhemostatic actions is direct or indirect cell signaling via TF-FVIIa or downstream coagulation proteases [38]. The metastasis process requires a proteolytically active TF-FVIIa complex [47].

The presence of mutations in the *F3* gene (Table 1) suggests the possibility of dysregulation of TF, leading to disrupted signaling pathways and cellular responses [48, 49]. This dysregulation may potentially be associated with altered TF expression and activity, may contribute to increased angiogenesis, metastasis, and therapy resistance in certain cancers like pancreatic, breast, and lung cancer. These mutations might have the potential to disrupt TF's interactions with coagulation factors and signaling molecules, causing imbalances in pro-coagulant and pro-inflammatory processes within the tumor microenvironment. Investigating TF mutations in cancer biology holds promise for developing targeted therapies to disrupt TF-mediated pathways, potentially benefiting patient outcomes.

**Table 1:** TF mutation status in cancers from the Cbioportal database (www.cbioportal.org)

Cancer type	Protein change	Mutation	Mutation type	COSMIC sample ID
Head and Neck Squamous Cell Carcinoma	F3-C11ORF80	-	Fusion	TCGA-D6-6517–01
	F3-C11ORF80	-	Fusion	TCGA-F7-A620-01
	I70V	1:g.95005817 T > C	Missense	TCGA-UF-A71A-01
	X251_splice	1:g.94997876C > T	Splice_Site	TCGA-CR-7399–01
Serous Ovarian Cancer	P238R	1:g.94997915G > C	Missense	TCGA-23–2649-01
	K233N	1:g.94997929C > A	Missense	TCGA-04–1648-01
Glioblastoma Multiforme	X71_splice	1:g.95001720G > A	Splice_Region	TCGA-12–0775-01
	D93N	1:g.95001656C > T	Missense	TCGA-19–5956-01
Lung Squamous Cell Carcinoma	S174R	1:g.94998715G > C	Missense	TCGA-85–6560-01
	Y189C	1:g.94998671 T > C	Missense	TCGA-56–8504-01
Bladder Urothelial Carcinoma	L274V	1:g.94996084G > C	Missense	TCGA-XF-A8HE-01
Uterine Endometrioid Carcinoma	G141R	1:g.94998816C > T	Missense	TCGA-AX-A2HD-01
	C81F	1:g.95001691C > A	Missense	TCGA-AP-A0LM-01
	K181N	1:g.94998694C > A	Missense	TCGA-D1-A17Q-01
	E127D	1:g.95001552C > A	Missense	TCGA-D1-A17Q-01
	Q222H	1:g.94997962 T > G	Missense	TCGA-BS-A0UV-01
	L44*	1:g.95005894A > C	Nonsense	TCGA-AX-A05Z-01
	T87Rfs*3	1:g.95001672_95001673del	Frame_Shift_Del	TCGA-AX-A3FS-01
	N170T	1:g.94998728 T > G	Missense	TCGA-B5-A3FC-01
	A112T	1:g.95001599C > T	Missense	TCGA-B5-A3FC-01
	E215*	1:g.94997985C > A	Nonsense	TCGA-E6-A1LX-01
	X251_splice	1:g.94996151 T > C	Splice_Region	TCGA-EO-A22R-01
Lung Adenocarcinoma	L55*	1:g.95005860_95005861del	Frame_Shift_Del	TCGA-05–4396-01
	N114Y	1:g.95001593 T > A	Missense	TCGA-97-A4LX-01
Cutaneous Melanoma	N231T	1:g.94997936 T > G	Missense	TCGA-EB-A24D-01
	E2K	1:g.95007189C > T	Missense	TCGA-GN-A26C-01
	V260I	1:g.94996126C > T	Missense	TCGA-D9-A6EA-06
	E94G	1:g.95001652 T > C	Missense	TCGA-D9-A6EC-06
	R168K	1:g.94998734C > T	Missense	TCGA-D3-A8GM-06
	S129F	1:g.95001547G > A	Missense	TCGA-FR-A8YE-06
Diffuse Type Stomach Adenocarcinoma	F179Lfs*5	1:g.94998700del	Frame_Shift_Del	TCGA-CG-4465–01
Tubular Stomach Adenocarcinoma	A200T	1:g.94998030C > T	Missense	TCGA-HU-A4GQ-01
	L175I	1:g.94998714G > T	Missense	TCGA-ZQ-A9CR-01
Colon Adenocarcinoma	S174N	1:g.94998716C > T	Missense	TCGA-AA-3858–01
	Y135C	1:g.95001529 T > C	Missense	TCGA-5 M-AAT6-01
Rectal Adenocarcinoma	N43D	1:g.95005898 T > C	Missense	TCGA-AG-A002-01
	D177Y	1:g.94998708C > A	Missense	TCGA-EI-6513–01

Its (*) part of mutation's name. The asterisk (*) is often used to denote a specific type of mutation called a stop codon mutation or a nonsense mutation. A stop codon is a specific sequence of DNA that signals the end of a protein-coding region. When a mutation occurs at this stop codon, it can result in a premature termination of protein synthesis

### TF cytoplasmic domain

The TF cytoplasmic domain (TF-CT) plays an important role in non-coagulant signaling. In contrast to the cytokine receptor superfamily, TF lacks adapter motifs that bind to Janus kinase-signal transducer and activator of transcription effectors (JAK/STAT) [20, 50]. The role of the cytoplasmic domain of TF is accompanied by conflicting results. On the one hand, some studies reported TF-CT is involved in metastasis and suggest tumor metastasis is relevant to the formation of an active TF-FVIIa complex and also palmitoylation-induced phosphorylation of the TF-CT. On the other hand, some studies have concluded TF-CT is not required in TF-FVIIa intracellular signaling [28, 47, 51].

The asTF is a potent pro-angiogenic molecule that binds to both integrins α6β1 and αVβ3 non-proteolytically, increasing FAK, PI3K/AKT, and MAPK signaling [20]. Moreover, PAR2 activation by TF-VIIa stimulates cell migration via mechanisms involving TF-CT phosphorylation [44]. The association between TF, integrins and PAR-2 appears to be complicated and regulated by the TF-CT [51]. Because PAR-2 signaling activates TF-CT phosphorylation, a regulatory circuit between TF-FVIIa-mediated PAR-2 signaling and the TF-CT is proposed. In fact, TF's tail phosphorylation regulates PAR-2 function in a reciprocal manner. However, the results of the TF's tail loss vary among different tumors [38].

## The role of TF in signaling pathways

### The role of TF in TF-VIIa-induced signaling pathways

It is TF that promotes the active formation of both the active protease FVIIa and the active zymogen FVII. In the absence of TF, VIIa shows little activity, due to specific sequence properties that keep VIIa in a zymogen-like state. The catalytic function of VIIa in vivo depends entirely on the presence of the TF-FVIIa complex because VIIa only develops full catalytic activity in the presence of the TF-FVIIa complex [52]. The interactions of TFs with its ligand Numerous physiological functions are influenced by FVIIa signaling pathways. Studies have shown that FVIIa's binding to TF affects a wide range of other important biological processes, including angiogenesis, embryonic vascularization, and tumor metastasis [47]. It was reported that TF-FVIIa-induced signaling in so many different cell types has the ability to alter the expression of genes that are responsible for encoding transcription factors and growth factors [53]. The TF- FVIIa signaling may play a role in cell survival, which is crucial for tumor growth and metastasis [38]. It has been demonstrated that the existence of TF-FVIIa complex causes cellular signaling events such as calcium fluxes, p44/42 mitogen-activated protein kinase (MAPK) phosphorylation, p38 MAPK phosphorylation, protein kinase B phosphorylation, up-regulation of several genes namely, early growth response gene-1, Cyr61, and connective tissue growth factor gene [29]. Formation of the TF-FVIIa complex alters the cellular physiology of the TF-expressing cell, notwithstanding the molecular mechanism not identified certainly [39]. For signal transduction, the TF-FVIIa complex is required to be proteolytically active [47]. Similar to the start of coagulation, a GPI-anchored TFPI controls the ternary complex's signaling activity. Caspase -3, one of the major caspases that initiate apoptosis and DNA degradation, was inhibited in the downstream pathways of the TF-FVIIa signaling [38]. Understanding how the TF-FVIIa signaling stimulates tumor progression can have useful therapeutic implications [47]. TF-FVIIa induces MAPK signaling via PAR2, as well as Src family kinase activation and activation of other pathways such as, PI3K, Janus kinase (JAK/STAT) [20]. A Recent study have identified an innovative proteolytic pathway through which TF-FVIIa modulates cellular interactions and tissue organization following coagulation stimulation. Notably, Eph receptors have been identified as novel targets for the proteolytic activity of TF/FVIIa. This proteolytic activity results in the cleavage of the Eph RTK (Receptor Tyrosine Kinase) family of receptors by TF-FVIIa [54].

### The role of TF in PARs dependent signaling pathways

TF also promotes cancer progression by activating signaling effects through a set of G-protein coupled receptors. TF signaling through Protease-activated receptors( PARs) plays a crucial role in the progression of multiple cancers [46]. The activation of PARs is the major signaling mechanism of the TF-FVIIa complex [51]. And a key role as an FVIIa docking site in order to the PARactivation is considered for TF in the cellular signaling process [29]. The binary TF-FVIIa complex activates PAR2 only at relatively high concentrations of VIIa, but at subnanomolar concentrations of VIIa, signaling of the TF-VIIa-X complex occurs [44]. PARs are members of G protein-coupled receptors (GPCRs), which are a large family of proteins with seven transmembrane domains [52]. Proteases of the coagulation cascade have the main role in activating PARs. The ternary complex (TF-FVIIa-FXa) formation is able to activate both PAR1 and 2 efficiently [52]. Both PAR1 and PAR2 signaling are strongly associated and can potentially activate each other. Trypsin-like serine proteases that recognize and cleave a particular arginyl peptide are the primary mechanisms by which the PARs are activated [29]. PARs are triggered by N-terminal proteolytic cleavage in contrast to classical receptors. When particular N-terminal peptides are removed, the resultant N-termini operates as tethered activation ligands, interacting with the extracellular loop 2 domain and beginning the receptor signaling [55]. PARs are members of a wide family of GPCRs that activate numerous signaling pathways after interacting with heterodimeric G proteins. These findings indicate that TF-PAR2 signaling in tumor cells is essential for tumor growth and that anti-TF approaches can be used in cancer therapy with only minor disruption of TF-dependent hemostatic pathways [45]. A variety of angiogenic regulators, chemokines, and anti-apoptotic genes that are associated with a wound-healing program are induced by PAR2 signaling [45]. The TF-FVIIa binary complex on tumor cells induces PAR2 activation, which in turn modifies the tumor microenvironment by causing a broad range of pro-angiogenic and immune-modulating cytokines, chemokines, and growth factors. It is still unclear how TFs and PARs (1 and 2) affect cell proliferation [56]. TF can organize the regulation of migration and cell adhesion in PAR2-dependent or independent ways [46].

### The role of TF in integrin signaling pathways

Integrins regulate many of TF's non-coagulant consequences. Numerous biological processes, such as migration, proliferation, and survival, are mediated by integrin. These transmembrane heterodimers serve as adhesion molecules for interactions between cells and with the extracellular matrix (ECM) [20]. Both signal-inducing and migration process is related to integrins [46]. The interaction of TF with integrins is constitutive in tumor cells and essential for TF-FVIIa-PAR2 signaling [56]. Point mutations in Lys-Gly-Glu (KGE) integrin-binding motif, which is located at the FVIIa protease domain lead to a decrease of TF-FVIIa association with integrins [57, 58]. Integrins facilitate TF-dependent PAR2 activation and signaling. Independent of PAR-2, direct TF binding to integrins also affects procedures like cell migration and signaling. Evidence has shown TF-dependent integrin signaling is involved in angiogenesis, and also TF –PAR2 signaling causes to produce of VEGF and pro-angiogenic molecules, including IL8, CXCL-1 [57].

Unlike flTF, asTF showcases a potent link with tumor size and grade and also triggers tumor growth and metastasis during both early and later stages of cancer by interacting with β1 integrins. asTF enhances the growth and expansion of tumors in a model of luminal breast cancer [59, 60]. HUTS-21 is an antibody that is specific for the β1 integrin subunit (membrane-proximal β-tail domain (βTD)), including residues 579–799 or a peptide simulating βTD. Reduced proliferation that is asTF-dependent but not flTF-dependent demonstrates the unique interaction of asTF with this β1 integrin region [60].

### The role of TF in MAPK, JAK, Src, PI3K, and Rac signaling pathways

The TF/FVIIa complex activates the three main MAP kinase family members, p42/p44 MAP kinase, p38 MAP kinase, and c-Jun N-terminal kinase (JNK), and causes calcium transients in different cell types [38]. P38MAP kinase and c-Jun N-terminal kinase are the members of the MAP kinase family and are critically dependent on the proteolytic activity of FVIIa for activation. Some studies have hypothesized that p38MAPkinase signaling induced by the TF-FVIIa is essential for the expression of MMP7, but the definite physiologic function of P38 MAP kinase is still doubted. Activation of MAPK phosphorylation is a crucial component in inducing cell migration [47, 50, 61].

Numerous intracellular signaling events involved in apoptosis are induced by the interaction of the TF and FVIIa, including activation of the PI3K/Akt and JAK/STAT5 pathways.because of considerable similarity with cytokine receptor class II family, like the members of this family, TF can activate STAT5 through JAK [51].

**Fig. 2:**
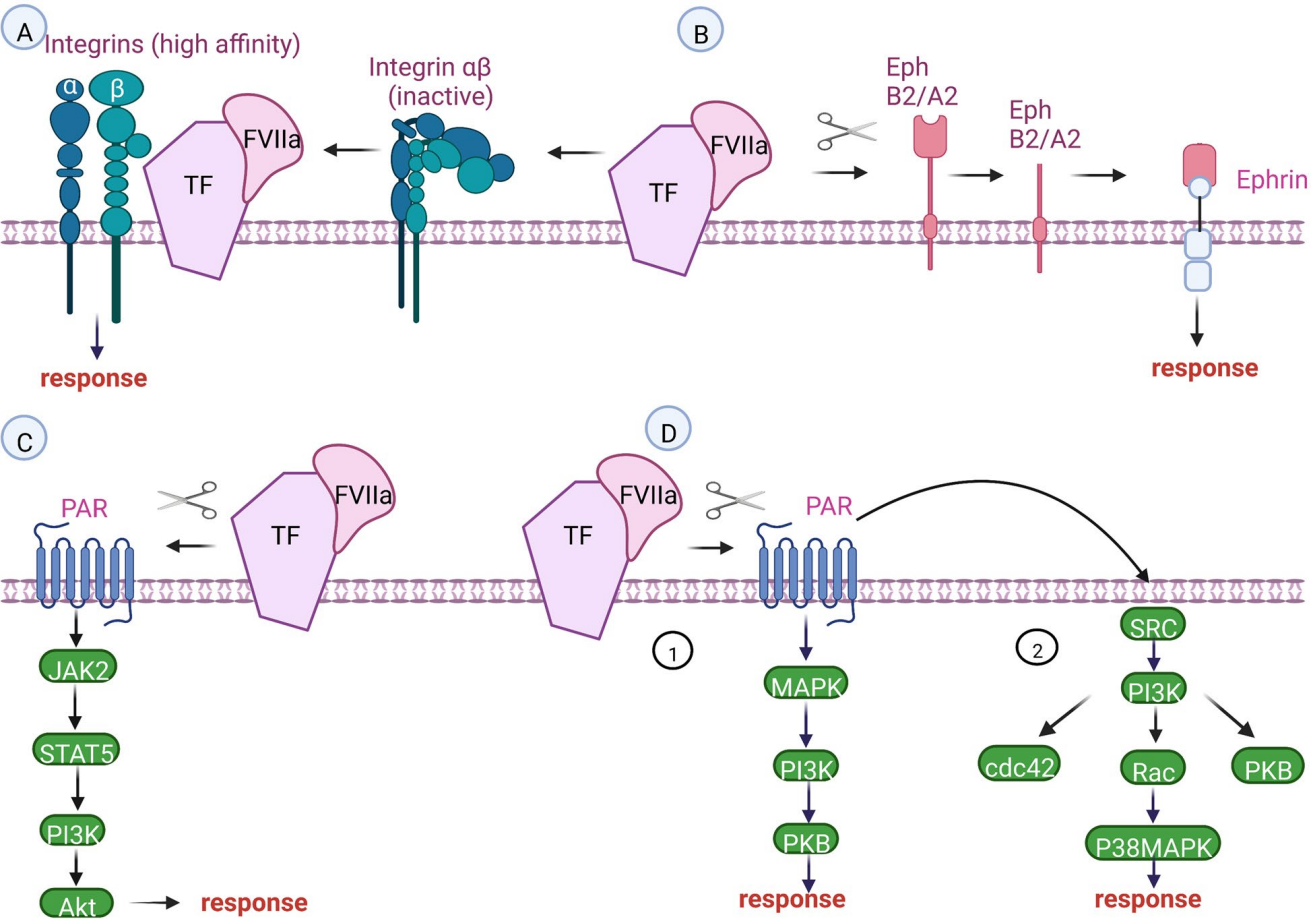
**A** Integrins: The binding of TF-FVIIa to integrin leads turning to a high-affinity condition for these molecules. This complex formation is independent of TF-FVIIa potency for cleaving PAR. **B** Ephs: TF-FVIIa binary complex can cleave both Eph B 2/ A 2 proteolytically; then, signal transduction is induced by ephrins. **C** JAK-STAT Proteolytic activity of FVIIa causes initiating of apoptosis-related signaling events such as activation of PI3K, Akt, by STAT5 phosphorylation. **D** 1-Function of the MAP Kinase and PI3K-PKB pathways due to the TF-FVIIa interaction through activating specific transcription factors in the nucleus leads to the gene expression and translation of the synthesized m RNA. **D** 2-The signaling pathway is activated by a binary complex that consists SRC-like family, PI3K, PKB, and Rac stimulation conducts cytoskeletal rearrangement

Important aspects of the TF's action in pathophysiology may be mediated by the association between TF signal transduction and the small GTPases of the Rho family. This family plays a significant role in controlling cell migration and motility. The interaction of TF and FVIIa activates a signaling pathway that includes the activation of Src-like family members. Consequently, leads to stimulating c-Akt/protein kinase B, which is a well-known downstream target of PI3K, as well as small GTPases Rac and Cdc42. Subsequently, the Rac activation directs P38 MAPK stimulation that results in cytoskeletal rearrangement (Fig. 2) [50].

## TF expression

There are many species of animals that contain this chemical, including humans, rats, fish, mice, Drosophila melanogaster, and insects [2]. Normal body tissues, such as vascular adventitia, capsules of various organs, tumor-related macrophages (TAM), endothelial cells, and fibroblasts, express TFs [62, 63]. In physiological conditions, TF is not detected in tuberculosis's mononuclear and vascular endothelial cells. However, in pathological conditions such as cancers, it is expressed on the surface of cancer cells [64, 65]. It has been shown that many pathological conditions, such as sepsis, myocardial infarction, arteriosclerosis, and cancer, express TF on the surface of monocytes, endothelial cells, and tumor cells [66]. Unlike benign breast tumors, malignant and invasive breast cancers express TFs on their surface near the endothelial cells of their stroma [67]. The amount of TF is low in the blood compared to the vessels of smooth muscle cells and fibroblasts adventitia [68]. An increase in the level of TF has been seen in prostate cancer [69]. Most tumors involving epithelial cells show increased TF expression [70]. TF plays a role in embryogenesis and is expressed in the embryo before factors VII and VIIa. Many cells temporarily or permanently express TFs in dead embryos and newborns [71]. Inflammatory conditions, tumor necrosis factor, tumor environment, etc., activate TFs. As a result, it leads to the progression of cancers such as ovarian, stomach, breast, etc. [65]. Therefore, TF is expressed on the surface of tumor cells and stromal cells [72].

The synthesis of TF is controlled by various factors, the most important of which are tumor suppressor genes, among which the most important genes are tumor protein p53 (TP53), phosphatase and tensin homolog (PTEN), and serine/threonine kinase 11 (STK11, also called *LKB1*). Previous studies showed that the expression of TF is controlled by TP53 and mutation in K Ras [73]. One of the factors that affect TF expression is post-transcriptional changes. Two important mechanisms are involved in these changes: microRNAs and alternative splicing phenomenon. These post-transcriptional changes control various biological activities such as invasion, metastasis, thrombogenesis, chemotaxis, growth, and angiogenesis in the tumor. Various factors such as hypoxia, VEGF, and pro-inflammatory cytokines such as TNF-α lead to increased expression of TF in tumors. Many factors are involved in the regulation of alternative splicing, such as the serine/arginine-rich (SR) proteins ASF/SF2, SC35, SRp40 and SRp55 or the Serine and arginine-rich (SR) proteins, Cdc2-like kinase (Clk)1, 2, 3 and 4, protein kinase B (Akt) or the DNA topoisomerase I (TopoI). The binding of SR proteins to TF pre-mRNA leads to the addition of exon 5 and, as a result, the production of flTF isoforms, but the reduction of binding of these proteins to TF pre-mRNA leads to the production of asTF isoforms [2, 74].

In a study [75] examining the regulation of TF isoforms in HUVECs, the impact of inhibiting the PI3K/Akt pathway and NFκB on TF isoform expression was compared. Inhibiting PI3K/Akt reduced asTF mRNA expression, while NFκB inhibition reduced both isoforms. The study also observed altered phosphorylation of SR proteins with PI3K/Akt inhibition. Overexpression of SF2/ASF and SRp75 influenced TF isoform expression. These findings highlight the role of the PI3K/Akt pathway in TF alternative splicing in HUVECs, independent of transcriptional regulation [75]. In another study investigating TF isoforms in TNF-α-stimulated human endothelial cells, the role of Cdc2-like kinases and DNA TopoI in TF splicing was examined. The study found that selective inhibition of these kinases led to changes in TF biosynthesis and impacted endothelial pro-coagulant activity [6]. This study provides the first evidence that serine/arginine-rich protein kinases modulate TF pre-mRNA splicing in human endothelial cells, influencing endothelial pro-coagulant activity during inflammation. The findings suggest a regulatory mechanism for TF isoform expression in response to TNF-α stimulation [6].

It was discovered that miR-19, a specific microRNA, directly regulates the expression of TF in breast cancer cells. By binding to the 3' untranslated region (3'UTR) of TF mRNA, miR-19 reduces the expression of TF. They observed higher TF levels in highly invasive breast cancer cells compared to less invasive cells, indicating the potential involvement of miR-19-mediated TF regulation in breast cancer invasiveness and angiogenesis [2]. It was also revealed that miR-93 and miR-106b also regulate TF expression in leiomyosarcoma cells through binding to the 3'UTR of TF mRNA. In 2013, it was demonstrated that inhibiting miR-19a or miR-126 increased the expression of flTF and asTF in human cells, resulting in enhanced pro-coagulant TF activity. This study provided the first evidence of miRNAs affecting TF isoform expression and function [2]. Furthermore, miR-19a can reduce TF expression in colon cancer cells, and inhibiting TF through miR-19a reduces migration and invasion of colon cancer cells mediated by TF [76]. These findings highlight the role of miRNAs in directly controlling TF expression and function in cancer cells, suggesting their involvement in cancer invasiveness, angiogenesis, and migration. Targeting specific miRNAs involved in TF regulation could have therapeutic implications for cancer treatment.

## The role of TF in apoptosis, metastasis, angiogenesis and VTE

**Fig. 3:**
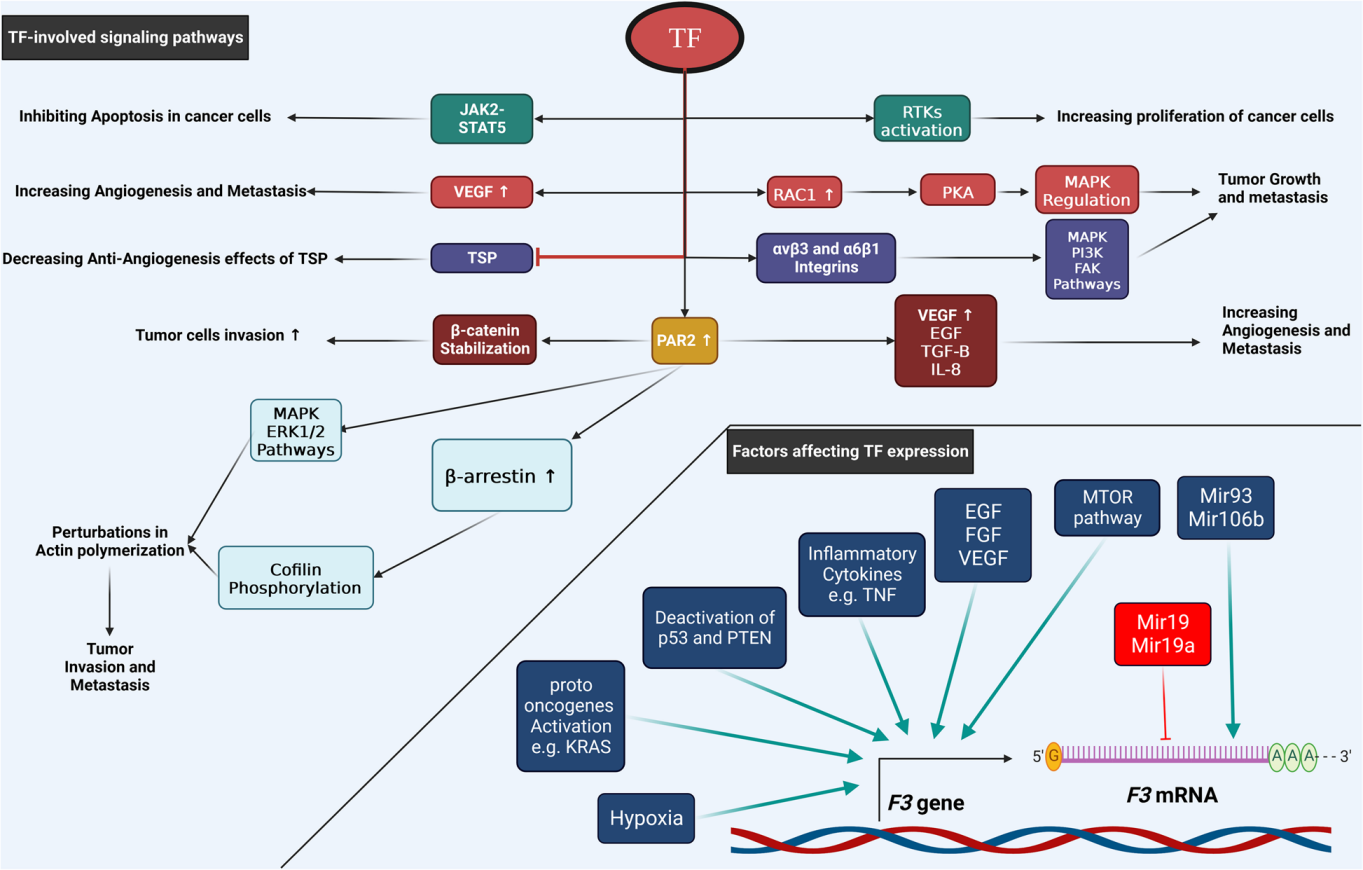
Overview on factors affecting *F3* gene and TF-involved signaling pathways progressing cancer. The right and bottom parts of the figure show the positive and negative effectors affecting the expression of TF gene, with blue arrows indicating positive effectors and red arrows indicating negative effectors. Activation of the mTOR pathway, inflammatory cytokines such as TNF, activation of proto-oncogenes such as KRAS, inactivation of PTEN and P53, growth factors such as EGF, FGF and VEGF, and hypoxia are positive effectors. On the other hand microRNAs such as miR19 and miR19a are negative effectors of the expression of TF gene. The top part of the figure shows how TF works in angiogenesis, invasion, metastasis, tumor cell growth, and carcinogenesis. On the one hand, TF leads to uncontrolled growth of cancer cells by activating the JAK2-STAT5 pathway, which prevents the apoptosis of cancer cells, and on the other hand, by activating RTKs, it promotes the growth of tumor cells. TF leads to increased angiogenesis and metastasis of tumor cells by increasing VEGF and inhibiting TSP. TF activates rac1, which in turn activates PKA and regulates MAPK, leading to the growth and metastasis of cancer cells. TF activates PAR2, which in turn leads to increased angiogenesis, invasion, and metastasis of cancer cells in several ways. On the one hand, it leads to increased VEGF, bEGF, IL8, and βTGF- and, therefore angiogenesis, invasion, and metastasis of tumor cells. Moreover, it stabilizes β-catenin and causes invasion of tumor cells. PAR2 also activates MAPK and ERK1/2 pathways, and increases β-arrestin, which phosphorylates cofilin, leading to the polymerization of actin filaments at the edge of invading cells and thus increasing invasion and metastasis of tumor cells. TF activates the signaling pathways PI3K/AKT, MAPK, and FAK by binding to integrins α6β1 and β3vα

In various cancers, TF plays a role in progression, invasion, metastasis, and angiogenesis. One way in which it contributes to these events is by activating signaling pathways [66]. Angiogenesis is influenced by the TF proteolytic pathway, one of the mechanisms by which factor VIIa plays a role in signaling [66]. The expression of TF on the surface of tumor cells leads to an increase in the expression of VEGF and, as a result, an increase in the expression of angiogenesis. The domain that plays an important role in angiogenesis is the cytoplasmic domain of TF with 21 residuals [77]. The cytoplasmic domain of TF plays a role in activating MAPK, p38 and GTPase, Rac-1, and as a result, the migration of tumor cells. Therefore, the inhibition of p38, for example, by SB20358, can lead to the inhibition of cell migration. Activation of the MAPK pathway by cytokines and growth factors leads to the enhancement of chemotaxis in vitro and angiogenesis in vivo. Phosphorylation of p38 leads to the activation of Rac1, and the activation of Rac1 leads to the activation of PKA, and finally, PKA leads to the regulation of MAPK. Phosphorylation of HSP-27 leads to p38 activation, F-actin polymerization, and as a result, cell migration. The activation of Rac-1 also leads to the activation of PI3K and is involved in cell migration. The cytoplasmic domain of TF plays a role in regulating the expression of VEGF in cell lines as well as calcium pumps in monocytes. For the activation of p42/44, p38, Rac-1, Src, PI3K, and p70/90 S6 kinase, only the extracellular domain of TF is required (Fig. 3) [66].

### TF-mediated metastasis

Metastasis is a multistep process that requires highly specialized interactions between tumor cells and host target organs [78]. To complete the multistep process of metastasis, tumor cells interact with the host in various highly specialized ways. This entails tumor cells entering the vascular compartment through local invasion, being arrested, and implanted in the target organs' capillary bed before proliferating as metastases [79]. The plasma coagulation cascades have long been considered to be essential for the efficient metastasis of tumor cells [80, 81]. In line with these findings, tumor cell production of pro-coagulants such as TF, cancer pro-coagulant 1, and selectin ligands corresponds with advanced disease and poor outcomes in numerous cancer types 2,3 [82]. An important regulator of intracellular signaling events, TF, regulates gene expression and cell function. It plays a key role in tumor cell intravasation, which is a crucial precursor to tumor dispersion. It has also been shown that a variety of hematological tumors are likely to spread based on their expression levels [81, 83].

TF may promote metastasis via promoting thrombin-induced proteolysis, intracellular signaling events mediated by the TF-CT, stimulation of protease-activated receptors by TF/FVIIa/FXa, or a combination of these activities. Endothelial cell protein C receptor (EPCR) overexpression or treatment with activated protein C (APC) decreases metastasis, but inhibiting endogenous aPC enhances metastasis linked with endothelial barrier disruption [84, 85]. Factor V Leiden, an aPC-resistant factor, causes enhanced hematogenous metastasis in mice [86]. These findings show that enhanced platelet activation may become a prominent pro-metastatic mechanism in prothrombotic situations. Several studies investigated the contributions of a crucial receptor interaction bridging platelets and leukocytes that have previously been involved in metastasis (i.e., GPIbα and CD11b) using pharmacological and genetic techniques [87–89]. According to N. Yokota et al. research, the contact pathway is not necessary for TF-dependent metastasis in TMPro (thrombomodulin deficiency) mice. Moreover, leukocyte contact with platelet GPIb is not necessary for increased metastasis in TMPro mice, and increased metastasis in TMPro mice is also a result of PAR1 signaling [89].

Yu et al. have revealed that tumor cells generated with activating K-ras or p53-inactivating mutations have enhanced TF expression. RNAi knockdown experiments in these cells revealed that TF upregulation is a major mediator of the K-ras mutation's tumor growth-promoting and pro-angiogenic actions [90]. Activated proteases produced by TF activity, including as FVIIa, Factor Xa (FXa), and thrombin, can activate PARs [91–93]. PAR signaling can activate NF-ĸB, suppress anoikis, and enhance the growth and metastasis of PAR-expressing cells, including tumor cells [94]. PARs, particularly tumor cell-expressed TF-FVIIa-PAR2, are implicated in the production of pro-angiogenic factors such as VEGF and IL-8, as well as immunologic modulators such as GM-CSF and M-CSF, thereby promoting tumor growth, increasing angiogenesis, and promoting metastasis [56].

Factor VIIa (FVIIa), is related to β1, β3, α6 integrins, but it is not related to α2 integrins and is independent of the proteolytic activity of Factor VIIa or Par2 signaling [45]. Proteolytic activity of Factor VIIa, is required for activation of Par2, not the extracellular domain of TF. The inhibited active site of Factor VIIa (FFR-VIIa), also plays a role in signaling. FFR-VIIa, through fibronectin in the lower part of Boyden's chamber, leads to cell migration, but FFR-VIIa alone cannot play the role as the chemoattractant agent. Because VIIa and FFR-VII bind to the inactive TF pool with different affinities but with the same affinity to the active TF pool, the active TF pool plays a role in cell migration [66].

It has also been reported that TF induces the formation of a platelet clot around tumor cells [95]. This platelet clot then stimulates the recruitment of macrophages to tumor cells, which are necessary for tumor cell survival. As a result, tumor cell TF can contribute to the metastatic process by initiating a biological mechanism that leads to macrophage recruitment to the tumor cell. Coagulation is also necessary for the recruitment of a comparable group of monocytes/macrophages to distant regions where circulating tumor cells establish themselves and grow. The substantial reduction in lung metastasis provided by interrupting this sequence of events brings up an intriguing therapeutic and potentially preventative opportunity in metastasis treatment [82]. In addition to these findings, platelets and fibrinogen have both been found to promote metastatic potential by reducing the ability of natural killer (NK) cells to remove newly formed micrometastatic foci [96, 97].

First demonstrated in 1990, Gorlin et al. found a molecular relationship between TF and actin-binding protein 280 (ABP-280) [98], which is responsible for the stability and mobility of the cortical actin cytoskeleton [99]. By recruiting ABP-280 to TF-mediated adhesion interactions, Ott et al. demonstrated that TF promotes cell adhesion and migration [77]. ABP-280 has been hypothesized to associate with other β2-integrin subunits due to sequence conversation in their cytoplasmic domains based on its association with the β-integrin subunit [100].

One of the factors that play a role in cell migration is the binding of ABP280 to the cytoplasmic domain of TF, which leads to the stabilization of actin filaments. In fact, this is caused by the interaction of the cytoplasmic domain of TF with the carboxyl-terminal of ABP280. ABP280 is activated by TNF-α and lysophosphatidic acid and regulates protein kinases activated by stress (such as MAPK). Induction of actin filament assembly following binding with TF leads to the phosphorylation of FAK (a non-tyrosine kinase receptor involved in cytoskeletal signaling). Extracellular accumulation of TF can identify cytoplasmic actin networks without dependence on integrin activity. Extracellular ligation of TF leads to the recruitment of ABP280, which is a necessary event for cell migration, indicating that TF is directly involved in the adhesion and migration of tumor cells. TFPI and Kunitz-type TF inhibitors lead to the inhibition of TF and, as a result, reduce cell migration and metastasis. It should be noted that cell migration takes place through the binding of ABP280 to the cytoplasmic domain of TF without dependence on the proteolytic activity of factor VIIa [66].

The binding of the protein ABP-280 to the cytoplasmic domain of TF is one molecular pathway through which TF may facilitate cell migration and trafficking. The recruitment of ABP-280 causes actin filament remodeling, cell spreading, and migration [101]. These effects are mediated by interactions of the TF cytoplasmic tail with cytoskeletal adaptor proteins, which may explain the TF-CT's functional role in metastasis and vasculogenesis.

A study revealed that microvesicles expressing TF that originated from tumor cells led to an increase in metastasis in mice through increasing the coagulation pathway and monocytes derived from the bone marrow. EVs expressing TF secreted from breast and pancreas cell lines, after 6 h of contact with endothelial cells, lead to the activation of endothelial cells and this activation requires TF activating factor X. The response of endothelial cells was mediated by Par1, not Par2. Therefore, Par1 is the main receptor of EVs expressing TF secreted from tumor cells in dormant and inactive endothelial cells [70].

PAR2 leads to an increase in the production of microvesicles (MVs) which have pro-coagulant properties, in the breast cancer cell line known as MDMA-MB-231. Rab-5, which is located in the cell membrane, plays an important role in MV production. In fact, PAR2, by phosphorylating and activating AKT, leads to the conversion of Rab-5-GDP to Rab-5-GTP and activates it. With the activation of Rab-5, actin polymerization occurs and leads to the release of MVs. Therefore, Rab-5 plays a role both in the release of MV and in the metastasis and invasion of cancer cells [102]. Most of the tumors involving epithelial cells show increased expression of TF. Endothelial cells express all 4 types of PARs; however, there is no consensus on the expression of PAR4 on the surface of umbilical cord vein endothelial cells in different studies. Par1 is highly expressed on the surface of inactive and dormant endothelial cells, while Par2 is only expressed on the surface of endothelial cells activated by inflammatory cytokines and hypoxic conditions. Endothelial cells in a normal state, do not allow the metastasis of cancer cells, but inflammatory mediators and cytokines lead to the increase of adhesive molecules and the release of inflammatory mediators from the endothelial cells themselves, and in this case, these activated endothelial cells allow Cancer cells metastasize. The complex of TF-VIIa-Xa expressed on the surface of microvesicles secreted from cancer cells, leads to the activation of endothelial cells by breaking Par. The response of activated endothelial cells to Xa is mainly carried out by PAR2. Increasing the expression of adhesive molecules such as E-selectin on endothelial cells can lead to the strengthening of the rolling phenomenon and increase the metastasis of cancer cells. In addition to E-selectin, interleukin-8, ICAM-1 and monocyte chemoattractant protein-1 lead to increased metastasis of cancer cells in response to extracellular vesicles (EVs) [70].

### TF-mediated angiogenesis

**Fig. 4:**
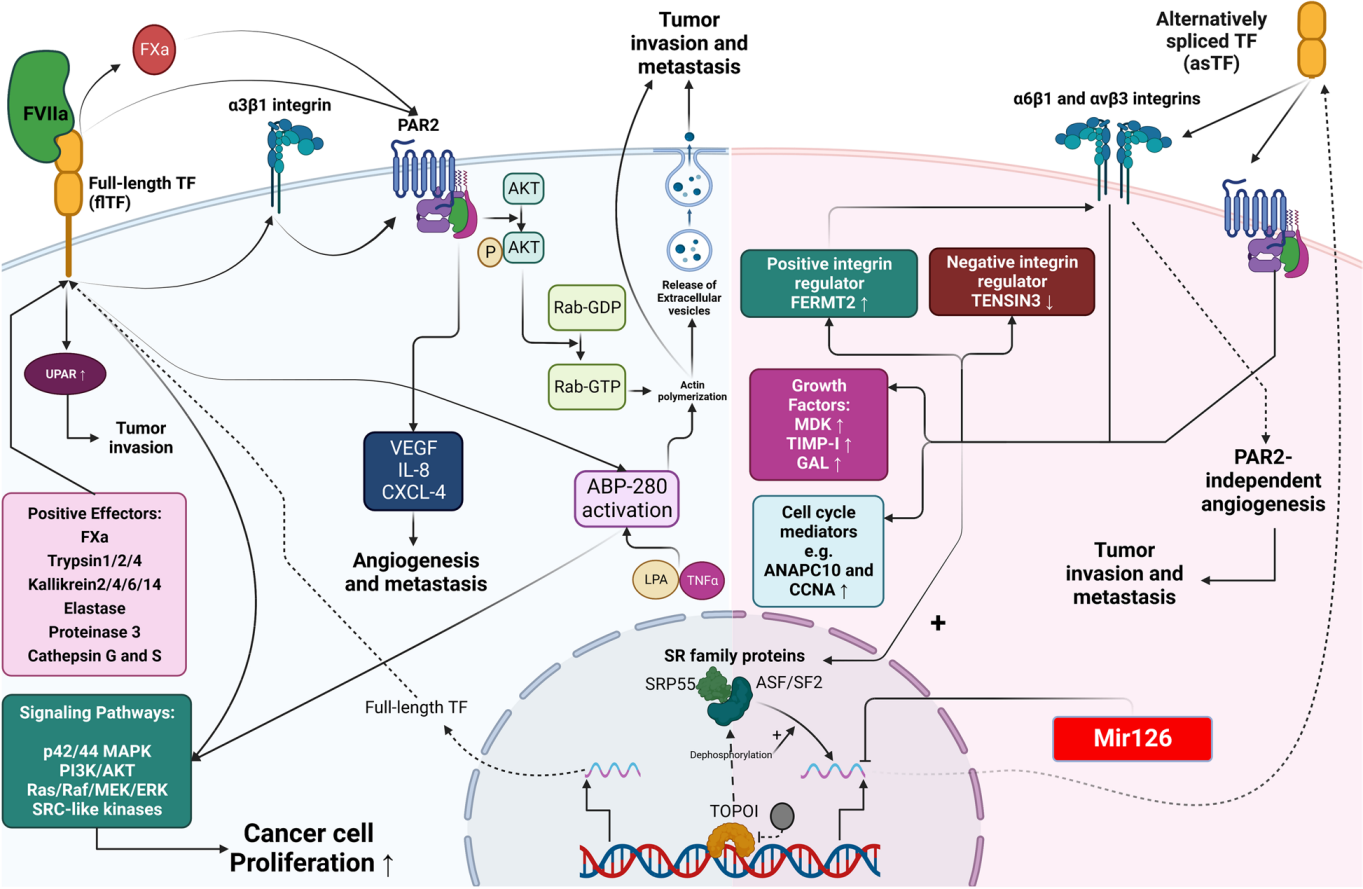
This text discusses the formation and role of flTF and asTF in carcinogenesis, as well as the roles of flTF -VIIa in the growth, invasion, and metastasis of cancer cells. The binding of TF to SR leads to the formation of asTF, while lack of binding leads to the formation of flTF. On the one hand, asTF increases cell cycle proteins (CNNA1/2 and ANAPC10), growth factors (MDK, TIMP-1, Gal), and factors involved in positive integrin regulation (FERMT2), while decreasing factors involved in negative integrin regulation (TENSIN3). Furthermore, binding to integrins α6β1 and β3vα leads to PAR2-independent signaling and consequently increased tumor cell metastasis. Decreased phosphorylation of SRP55 and ASF/SF2 following TOPOI inhibition leads to increased asTF expression and production, while miR126 results in decreased asTF expression and production. The binding of flTF to integrin α3β1 and increased PAR2 expression leads to increased VEGF, IL8, and CXCL1 expression, angiogenesis, and increased tumor cell metastasis. miR19a decreases flTF production and expression. flTF-VIIa plays a role in carcinogenesis in several ways: **1**) increasing UPAR expression and consequently tumor cell invasion, **2**) phosphorylation and activation of AKT following PAR2 activation, which results in the conversion of inactive Rab-GDP to active Rab-GTP, actin polymerization, and release of microvesicles (MV), ultimately resulting in tumor cell invasion and metastasis, and **3**) increasing cancer cell growth following activation of the P42-P44 MAPK, PI3K/AKT, RAS/RAF/MEK/ERK, and SRC-like kinase signaling pathways. flTF, via binding to ABP-280, which is itself activated by TNF-α and lysophosphatidic acid (LPA), can activate the aforementioned signaling pathways. The flTF-VIIa complex, binding to factor Xa, leads to PAR2 cleavage and activation of endothelial cells. Factor Xa, trypsin 1/2/4, kallikreins 2/4/6/14, elastase, protease 3, and cathepsins G/S activate PAR2, while thrombin, Xa, and APC cleave and activate PAR1, which all act in favor of tumor progression and invasion

Angiogenesis, the phenomenon through which new blood vessels form from already-existing ones, occurs during embryonic development, wound healing, and cancer [103]. Vascular endothelial growth factor (VEGF), growth factor receptors like KDR/flk-1 and flt-1, metalloproteinases, and interleukin-8 are just a few of the proteins that control angiogenesis; however, integrins are also necessary. In particular, capillary formation and endothelial and pericyte migration are regulated by β1 and β3-type integrins [104, 105]. It has been noted that Angiogenesis has been identified as a defining feature of cancer and has been shown to be a requirement for tumor growth [106, 107]. Tumor growth and angiogenesis are thrombin-independent and involve TF-CT-mediated cell signaling, activation of the protease-activated receptor (PAR) 2, and integrin ligation (Fig. 4) [40, 45, 63].

In the biology of cancer, flTF and asTF have important roles in thrombogenicity, survival, tumor growth, angiogenesis, signaling, invasion, and metastasis [1]. Hobbs et al. demonstrated that asTF overexpression increased the microvascular density in a pancreatic cancer tumor model and hence facilitated cancer-related angiogenesis in vivo. They injected mice with pancreatic MiaPaCa-2 cancer cells that were overexpressed with asTF. These studies revealed that asTF overexpression increased the number of tumor-associated blood vessels, tumor cell proliferation, and, ultimately, in vivo tumor growth [108]. Eisenreich et al. showed that overexpression of asTF improved the pro-angiogenic potential of both A549 lung cancer cells and murine HL-1 cells [1], which is in line with previous studies that reported asTF mediate pro-angiogenic processes. They showed that asTF overexpression accelerated the growth of A549 lung cancer cells. Additionally, Eisenreich et al*.* discovered that endothelial cells formed pro-angiogenic tubes in response to asTF secreted by A549 cells [7, 109]. These mechanisms, which were unrelated to flTF, were linked to enhanced production of pro-angiogenic and cell growth-promoting substances such as cystein-rich 61 or VEGF [7]. In 2009, van den Berg et al. showed that asTF-induced pro-angiogenic processes in endothelial cells were mediated by integrin ligation, which was different than PAR-2 signaling. In this situation, they discovered that integrin ligation was the mechanism through which asTF-induced angiogenesis was mediated. This was not dependent on PAR-2 or FVIIa, in contrast to flTF-mediated angiogenesis [40].

TF overexpression in tumors contributes to the angiogenic phenotype in part by up-regulating the expression of the pro-angiogenic protein VEGF and downregulating the expression of the antiangiogenic protein thrombospondin-2 [110]. In vivo, TF may stimulate local thrombin production and hence indirectly induce VEGF signaling via paracrine PAR1 signaling in stromal cells or autocrine PAR1 activation of tumor cells [111]. Transfectants carrying the extracellular domain mutant TFmut, which has noticeably reduced activity for activating factor X [112], generated basically similar amounts of VEGF mRNA as those carrying the full-length TF cDNA [101]. According to the findings so far, it is not necessary for TF pro-coagulant action and binding factor VIIa's proteolytic function to regulate VEGF synthesis in human tumor cells [113]. About equal amounts of VEGF mRNA were produced by transfectants with the extracellular domain mutant and the full-length sequence. Nevertheless, cells transfected with the mutant cytoplasmic deletion construct generated more TF but little to no VEGF. Therefore, in some tumor cells, the cytoplasmic tail of TF regulates the expression of VEGF [101].

VEGFR-1, also known as FLT-1, is a transmembrane tyrosine kinase receptor for VEGF-A, VEGF-B, and placental growth factor (PlGF). In vitro studies have shown that VEGFR-2, unlike VEGFR-1, is the only receptor required for endothelial cell proliferation, migration, and survival [114–116]. VEGFR-1 signaling has also been shown to play a beneficial regulatory role in other studies. The reduction in PlGF expression in ischemic myocardium is linked to decreased angiogenesis, suggesting that VEGFR-1 or VEGFR-2 heterodimerization is required for angiogenesis [117]. In neonatal and adult mouse tissue, Vivienne C. Ho and Guo-Hua Fong observed that Cre-loxP-induced deletion of VEGFR-1 enhanced angiogenesis [118]. However, VEGFR-1 has been implicated in stimulating angiogenesis under certain circumstances in adult tissues, especially in tumors and ischemic tissues, despite negatively controlling endothelial cell differentiation throughout development.

In areas of hypoxia within solid tumors, hypoxia-inducible factor-1 (HIF-1a) is recognized to increase VEGF expression. Although TF might increase VEGF expression, oxygen concentration drives angiogenesis [119]. In endothelial cells, microRNAs are involved in post-transcriptional changes. These post-transcriptional changes control various biological activities such as invasion, metastasis, thrombogenesis, chemotaxis, growth, and angiogenesis in the tumor.

### TF-mediated venous thromboembolism

One of the cases that occurs in various cancers is VTE, which is a common disorder and one of the leading causes of death in patients with brain tumors, so about 34% of brain tumor sufferers afflict VTE during their disease process [120–123]. There is no significant relationship between TF expression and increased risk of VTE in the future [120]. Microparticles expressing TF, which are the cause of thrombosis and platelet aggregation in some cancers, are released in a small amount from the cells in the brain tumor [124]. It is also stated that low-grade astrocytomas express a higher level of TF compared to the previous study, and it is also stated that astrocytes are considered the primary source of TF in the central nervous system of mice [125–127].

One of the factors that lead to VTE in various cancers is the release of microvesicles (MV) expressing TF from cancer cells. In general, we have two types of extracellular vesicles: 1. The smaller exosome, which has a size between 30 and 100 nm, is formed in the cell and is released after merging with the cell membrane 2. The larger microvesicles, which have a size between 50 to 1000 nm, are formed directly by budding out of the plasma membrane. Many studies showed that microvesicles containing TF that are secreted from tumor cells would lead to VTE. Because microvesicles sprout from the cell membrane, they have the cell's characteristics from which they sprouted. The number of microvesicles is dependent on age, so their number decreases with age because, with aging, microvesicles are more easily absorbed by B cells. Microvesicles lead to the activation of monocytes and B cells, and the stimulation of B cells with LPS leads to the absorption of MVs into these cells. Therefore, if a middle-aged person has a large number of MVs, it may occur due to a disease. Inflammatory cytokines and endotoxins will increase the release of MVs from tumor cells. There is a direct relationship between ERK phosphorylation and TF activity. Larger microvesicles express more TF and, as a result, increase the risk of VTE [128]. Mackman et al*.* have reported that microparticles (MPs) expressing TF originates from tumors and could be a good biomarker for reconnoitering patients at risk for venous thrombosis. They also reported that if a patient develops cancer and has VTE concurrently, the activity of MP expressing TF will be much higher than cancer patients without VTE. So they concluded TF^+^ MPs might be a useful biomarker for reconnoitering cancer patients and other patients who have a high risk for venous thrombosis [129].

### TF-mediated activation of anti-apoptotic pathways

In addition to TF’s role in the process of coagulation, angiogenesis, metastasis, and invasion of tumor cells, It also plays an anti-apoptotic role. Apoptosis, or programmed cell death, has two internal and external pathways in which caspases are involved in both pathways. Caspases are normally inactive (zymogen) and are activated by proteolytic cleavage. The extrinsic pathway is activated by death receptors such as FAS, TRAIL, and TNFR1, leading to the activation of caspases 8, 10, and 12, which ultimately activate caspase 3. The internal pathway is activated following oxidative stress, deprivation of growth factors, etc., which leads to the release of cytochrome c from mitochondria. These events will eventually lead to the activation of caspase 9 and 3. Studies have shown that the binding of VIIa to TF, leads to the prevention of apoptosis in cells following growth factor deficiency and other factors, and the anti-apoptotic protease activity of VIIa is dependent on its binding to TF [130, 131].

This anti-apoptotic activity of the complex of TF and VIIa is due to the activation of signaling pathways that are involved in cell growth and survival, including the p42/44 MAPK pathway, which is activated by phosphorylation. The PI3K/AKT pathway, the RAS/RAF/MAPK/ERK pathway and SRC-like kinases. There are conflicting studies regarding the association of active factor VII with PAR in the process of inhibiting apoptosis. Previous studies have stated that VIIa cannot lead to an increase in intracellular calcium release in BHK (baby hamster kidney) and CHO (Chinese hamster ovary) cells, as well as its insensitivity in desensitization of cells to PAR activators, it can be concluded that intracellular signaling Active VIIa, does not depend on PAR. One of the reasons that VIIa is not able to release intracellular calcium is that because the activation of PAR is kinetically slow and requires the binding of VIIa to TF, the release of intracellular calcium cannot be achieved by VIIa and PAR. TF leads to increased survival of breast cancer cell lines, namely MDA-MB-231 and ADR-MCF-7 [130, 131]. In ADR-MCF-7, the TF-VIIa-Xa complex and not the TF-VIIa complex is necessary for survival, and the p42/44 MAPK pathway is necessary for the survival of MDA-MB-231.

The activated JAK2/STAT5 pathway, following the effect of TF, plays an important role in inhibiting apoptosis. STAT due to its effect on the anti-apoptotic protein BCL-XL, and JAK by its effect on AKT, leading to the inhibition of apoptosis. TRAIL leads to apoptosis through the formation of the DISC complex and the subsequent activation of caspase 8 and 3. Some studies have shown that the cytoplasmic domain of TF is not necessary for the formation of the TF-VIIa complex, nor for the activation of coagulation pathways. The TF-VIIa- Xa complex leads to the induction of signaling pathways through PAR1 and PAR2, especially at low concentrations of VIIa. Therefore, by using PAR1/2 neutralizing antibodies, we understood that TF performs its anti-apoptotic activity without dependence on PAR1/2. PAR2-activating agonists do not decrease the expression of DAPK1 (a tumor suppressor and a calcium-regulating serine-threonine kinase) [130, 131].

VIIa leads to the phosphorylation of transcription factor STAT5 by Janus kinase 2 (JAK2) and the heterotrimer of G protein family, that is, G12/13. Gi has no role in this process. In fact, the activation of STAT5 is caused by the proteolytic activity of VIIa and has nothing to do with the activation and mediation of Xa, thrombin, and also with the cytoplasmic domain of TF. The noteworthy point is that although the activation of JAK1, JAK2 and TYK2 may be seen simultaneously with the phosphorylation of STAT5, only JAK2 plays a role in the phosphorylation of STAT5. STAT5/JAK2 signaling, following the proteolytic activity of VIIa, leads to the production of anti-apoptotic protein BCL-XL, and thus leads to increased cell survival. Likewise, JAK2 leads to the activation of another anti-apoptotic kinase called PKB, and the activation of this kinase following JAK2 is regulated by the PI3K signaling pathway [132]. Phosphorylation of JAK1/2 and STAT5 occurs at a low concentration of 10 nmol/liter, but the proteolytic activity of VIIa and subsequent activation of the Akt/PKB signaling pathway occurs at a higher concentration, i.e., 10 nmol/liter. Through JAK2 inhibitors such as AG490, JAK2 and subsequent phosphorylation of STAT5 can be inhibited, and as a result, the anti-apoptotic activity caused by these factors is also inhibited [132].

In addition to TF, Xa and thrombin can act as anti-apoptotic agents. Xa and thrombin in BHK cells expressing TF, lead to inhibition of apoptosis. In two cell lines BHK (baby hamster kidney) and CHO (Chinese hamster ovary) that express TF, the signaling pathways of p42/44 MAPK and AKT are activated. If PI3K inhibitor like LY294002 and also MEK inhibitor like U0126 are used, VIIa can no longer exert its anti-apoptotic effects. The expression level of DAPK1 (death-associated protein kinase 1) mRNA was changed by the TF/VIIa complex. DAPK1 sensitizes cells to many apoptotic signals and blocks signals involved in tumor cell metastasis. So, the anti-apoptotic activity of the TF/VIIa complex is carried out through the regulation of the expression of caspase-8 and DAPK1 and without dependence on PAR1/2 and through the PI kinase/AKT signaling pathway [130, 131].

VIIa does not need thrombin and Xa for its activity and is inhibited by the active site blocker of VIIa (VIIai). VIIa in physiological concentration, leads to the inhibition of caspase 3 and as a result, the absence of apoptosis in cells expressing TF but not in cells that do not express TF. G protein of class 3, called Gq, which is activated by thrombin, leads to increased calcium release and, as a result, the beginning of calcium-mediated signaling. The blocking effect of the active site blocker of VIIa (VIIai), depends on the concentration and at a concentration of about 10 times the normal concentration, it leads to the inhibition of cell survival dependent on VIIa. The effect of VIIa is stronger in cells in adherent environments, and in these environments, the need for VIIa to transmit the survival signal is greater. VIIa plays a role in the anoikis of tumor cells of blood origin. VIIa leads to the activation of phosphatidyl inositide 3-kinase (PI3K) and p42/44 MAPK signaling pathways. Increased expression of TF, up to 1000 times, is characteristic of metastatic cells. The TF-VIIa complex leads to the activation of p21 RAS. VIIa leads to the activation of the PKB pathway in cells expressing TF, and The TF-VIIa leads to an increase in interleukin-8. The TF-VIIa, either in serum-free cells (Starved serum) or in non-adherent cells, will lead to increased cell survival. The role of TF and VIIa in metastasis depends on their concentration so that the normal concentration of TF leads to increased survival, but VIIa in physiological concentration (10 nm) does not have this effect, but in a lower concentration, i.e., 1 to 5 nm leads to increased survival. The combined activity of TF and VIIa has a much stronger effect on cell survival than the activity of each of these two factors alone [132, 133].

## The role of TF in various cancers

### Pancreatic cancer

TF is not expressed in normal pancreatic cells, but its expression is increased in invasive or non-invasive pancreatic cancer cells. In pancreatic cancer, hemostatic activation is often observed, which may be due to angiogenesis and venous thromboembolism, which Sproul discussed in 1938 [134, 135]. A study also found that elevated TF expression was associated with symptomatic venous thromboembolism in pancreatic cancer specimens [136]. According to previous studies, TF leads to an increase in the expression of factors involved in angiogenesis, such as VEGF and a decrease in the expression of anti-angiogenic factors, such as thrombospondin, and in this way, they are involved in the invasion and metastasis of many cancers, including pancreatic cancer. The increase in TF expression leads to an increase in VEGF and microvessel density. TF overexpression in pancreatic tumors increases the occurrence of vascular thrombosis in this cancer by four times compared to normal (Table 2) [136].

**Table 2:** Overview on the effect of TF in various cancers

Cancer	Presence of TF	Result	Study
Human melanoma	TF overexpression	Overexpression of TF increased metastasis	[137]
Colorectal cancer	TF expression	Significant connections between TF expression and both synchronous and metachronous hepatic metastases	[138]
Breast cancer	TF expression	TF may have a role in neovascularization associated with tumor stroma formation and also patients with TF expression had lower survival periods than individuals without TF expression	[68, 139]
Hepatocellular carcinoma	TF expression	TF is a regulator of angiogenesis in HCC with VEGF also, TF plays a crucial role in the aggressiveness of HCC	[140]
Colorectal cancer	TF expression	Expression of TF caused cell line SW620 growth and migration	[141]
Non-small cell lung cancer	TF overexpression	TF staining was substantially greater in cells from NSCLC patients having metastasis than in those without metastasis based on immunohistochemical studies	[142]
Melanoma	TF overexpression	In a mouse model, higher levels of TF were present in metastatic melanoma cells than in nonmetastatic cells, and blocking TF receptors significantly decreased the number of melanoma cells that remained in the lung	[78]
Glioma	TF expressed	In this prospective cohort study, no strong association was found between TF expression levels in brain tumors and the occurrence of VTE	[125]
	TF overexpression	There is a significant correlation between TF expression and tumor micro-vessel density, a measure of angiogenesis, suggesting that TF may play a role in glioma angiogenesis induction. As a result, TF is highly expressed in gliomas and the degree of expression is correlated with the microvascular density and histologic grade of malignancy	[126]
Ovarian cancer	TF expression	A substantial correlation between TF expression in tumors and the development of VTE was found	[143]
Gastric Cancer	TF expression	TF expression was associated with lymph node metastases. It is hypothesized that TF expression is crucial in the metastasis of intestinal-type gastric cancer but not diffuse-type cancer	[144]
Retinoblastoma	TF expression	TF modulates retinoblastoma tumor angiogenesis. In the proliferative region of retinoblastoma, TF was preferentially expressed	[43]
Pancreatic cancer	TF overexpression	A significant correlation between elevated TF expression in pancreatic cancer specimens and symptomatic venous thromboembolism. Also, TF expression is a crucial early stage of the pancreas' malignant transformation	[136]
Prostate cancer	TF expression	Overexpression of TF in the majority (73%) of human prostate cancers	[145]
Lung carcinoma	TF expression	patients with lung carcinomas that were TF-positive had lower survival periods than individuals whose cancers were TF-negative	[146]

*TF* Tissue Factor, *HCC* Hepatocellular Carcinoma, *VEGF* Vascular Endothelial Growth Factor, *VTE* Venous Thromboembolism

It is possible that TF may play a role in tumorigenesis, perhaps by regulating angiogenesis, based on the high frequency of TF expression found in the Khorana study in both invasive and noninvasive pancreatic neoplasms. It has been suggested that TF-mediated signaling may play a crucial role in regulating angiogenesis [147]. There is evidence that tumor cells may release TF into the bloodstream and contribute to systemic thrombogenesis. Cancer patients have been found to have higher plasma TF levels, and experimental research has suggested that circulating TF may play a role in the formation of thrombi [148, 149]. Pancreatic ductal adenocarcinoma (PDAC) is a deadly tumor with low survival rates and associated thrombotic complications. Increased TF expression, driven by oncogenes and tumor suppressor inactivation, contributes to PDAC progression. asTF, expressed in early and advanced stages, activates signaling pathways and integrins, promoting metastasis, migration, and monocyte infiltration. Both host TF and asTF contribute to thrombus formation. PDAC cells with high asTF expression release MPs with pro-coagulant activity. [150].

Coagulation factors such as thrombin and fibrin lead to the attachment of tumor cells to platelets and endothelial cells. Because the TF leads to the initiation of the external process of coagulation and, subsequently, the production of thrombin and fibrin, it plays a major role in the progression of invasion and prognosis of many solid tumors, such as pancreatic ductal adenocarcinoma. Therefore, the use of drugs that inhibit thrombin, warfarin, and heparin with low molecular weight can increase the survival time of patients [151].

### Gastric cancer

The increase in fibrinogen plasma levels and thrombin formation in patients with non-metastatic gastric cancer increases their risk of thrombotic events [152–154]. According to Yamashita et al. study, approximately 25.1% of gastric cancer patients had significant levels of TF within their carcinoma cells. TF was present in 41.8% of total intestinal-type carcinomas and 61.0% of advanced intestinal-type carcinomas, significantly higher than diffuse-type carcinomas [144]. The rate of TF expression in overall gastric cancer is relatively low, but it was comparable to the results in colorectal cancer [155].

In the population with intestinal-type gastric carcinoma, TF expression was not associated with diffuse-type gastric carcinoma but rather with advanced disease, invasive features, and poor outcomes [62]. These results suggest that TF expression is essential for intestinal gastric cancer metastasis, not diffuse gastric cancer metastasis. In SGC-7901 gastric cancer cells, Zhang et al. transfected a full-length antisense TF cDNA, a shortened TF cDNA, an extracellular domain mutant cDNA of TF, and a vector containing flTF cDNA. Transfectants with the full-length and extracellular domain mutant TF cDNAs raised TF and VEGF levels, while transfectants with the truncated TF cDNA increased TF but had low VEGF levels. These findings suggest that the cytoplasmic tail of TF is involved in the synthesis of VEGF in human gastric cancers [156].

The expression level of asTF and flTF mRNA in gastric cancer tissues is significantly higher than the expression level of the two TFs isoforms mentioned above in healthy gastric tissues of healthy people. So, both TF isoforms (asTF and flTF) can be helpful predictors of prognosis in gastric cancer, and their level is vital in this cancer. Aberrant expression of some oncogenes, such as KRAS, can lead to aberrant expression of TF and subsequently cause cancer. Among other factors that increase TF expression and cancer progression include activation of epidermal growth factor receptor) EGFR), inactivation of p53, and PTEN tumor suppressor [41].

### Breast cancer

Increased expression of TF has been observed in more than 90% of breast cancers. The expression of TF on the tumor cells indicates a bad prognosis for breast cancer [72]. In addition to the TF, other risk factors that can affect the prognosis of the disease are age, size, the degree of tumor involvement, and hormone receptors. Estrogen, Her2, and nodal status. It is not clear whether TF predicts the prognosis of breast cancer or not [68]. Begum’s study showed asTF involved in breast cancer progression had low flTF expression regardless of its effect on angiogenesis. Also, asTF in the angiogenesis process in the xenograft model MDA-MB-231-mfp does not increase the expression of vessels expressing marker CD31 in the model 2A3-3 expressing asTF, indicates the role of asTF in angiogenesis of this model. As a result, this study showed that asTF is abundant in breast cancer and plays a role in its progression and proliferation [157].

TF expression was found in small vessel vascular endothelial cells in the stroma of invasive breast carcinomas but not in benign breast cancers. This finding is particularly significant since it implies that TF may have a role in neovascularization associated with tumor stroma formation [67]. Invasive breast cancers are characterized by an increase in TF-expressing cells in the stromal compartment. This was particularly apparent in the Comedo-ductal carcinoma in situ, where macrophages that expressed TF were plentiful. Inflammation in such tumors could be comparable to the inflammatory response that occurs during normal wound healing [158]. Ryde´n et al*.* studied the relationship between PAR-2 expression and TF phosphorylation in human breast cancer. They used xenograft tumors and found positive staining for TF. In PAR-2-deficient mice, tumors showed no cytoplasmic staining for pTF. The study highlighted the importance of TF-PAR-2 signaling in breast cancer prognosis and suggested pTF as a biomarker for deregulated TF-PAR-2 signaling in primary tumors [44, 159]. Versteeg et al*.* investigated the role of PAR1 and PAR2 in breast cancer development using PAR1-/- and PAR2-/- mice. They found that PAR1-/- breast cancer cells were unresponsive to thrombin, while PAR2-/- mice showed slower tumor progression and reduced metastasis. Additionally, vascularization and metastasis were delayed in PAR2-/- mice. The study concluded that PAR2, but not PAR1, signaling promotes the development of mammary adenocarcinoma [44, 160]. Therefore, the role of TF in the progression of breast cancer needs further investigation [68, 161, 162].

TGF-beta immunoreactivity was found in both tumor cells and the extracellular matrix surrounding TF-positive stromal cells after double immunofluorescent staining for TF and TGF-beta [67]. Because TGF-beta has been shown to be a strong chemoattractant for fibroblasts [163], to stimulate myofibroblast cytodifferentiation [164, 165], and to activate TF expression in myofibroblast indicator cells. Also, TGF-beta deposition in the stroma surrounding infiltrating tumor cells appears to contribute to the abundance of TF-expressing myofibroblasts in these regions. It was demonstrated for the first time that antibodies against TFs were capable of suppressing tumor growth in vivo [83]. The anti-TF antibodies have been shown to reduce metastasis in mice experimental models [78] but not initial tumor growth. A therapeutic level of CNTO 859 (anti-TF antibody) was also demonstrated to inhibit tumor incidence and growth in orthotopically implanted MDA-MB-231 cells.

### Prostate cancer

Prostate cancer is another cancer in which TF levels have increased. Almost 80% of people with untreated prostate cancer respond to androgen deprivation therapy. TAKUYA’s study has shown that TF expression in prostate cancer is one of the factors involved in the prognosis of this disease. It should be noted that in some previous studies, it has been stated that the TF level is not related to the patient's condition [69].

TF has been found to be overexpressed in most human prostate cancers (73%), suggesting it may have a functional role in prostate tumor development [145]. TF is exclusively expressed by malignant luminal epithelial cells, while the tumor-associated stroma exhibits minimal staining. TF has been shown to be highly expressed by stromal components associated with breast carcinoma, in contrast to this pattern of immunoreactivity. This immunoreactivity pattern resembles that seen in gliomas, lung, and pancreatic carcinomas [127, 146, 166], where TF is expressed by the malignant tumor cells themselves. Numerous adverse prognostic factors for prostate cancer, such as microvessel density, preoperative PSA levels, and positive surgical margins, were associated with TF expression. While one of the best indicators of prostate cancer recurrence is the preoperative PSA level, increased tumor microvessel density has been identified as a major unfavorable predictive factor in prostate carcinomas [167–169]. Therefore, determining the prostate cancers' TF expression status may offer further prognostic data. However, it should be highlighted that neither the Gleason grade nor the present patient condition showed a significant correlation with the TF status in Abdulkadlr et al. investigation.

To determine the link between TF and these factors, more data may be required. The relationship between TF and the malignant phenotype in prostate carcinomas raises the possibility that tumor cells can be specifically targeted in vivo using this cell surface receptor. Using TF antibodies or ligands linked to different toxins, it might be possible to destroy prostate cancer cells only when they are specifically targeted for destruction. Additionally, there is information that TF function suppression by itself can retard the growth of tumors. Mueller et al. exploited anti-TF antibodies to inhibit the transmission of human melanoma cells to other mice [78]. Abdulkadlr et al. found that TF is overexpressed in prostate cancer and associated with poor prognostic factors. Further research is needed to understand its role as a marker and its induction in cancer cells. Langer et al. examined preoperative plasma TF antigen levels in localized prostate cancer patients and their association with disease prognosis. Patients with high TF levels had increased platelet-derived microparticles and a higher risk of recurrence. Elevated preoperative plasma TF antigen levels might indicate increased platelet activation and a higher risk for recurrent illness in localized prostate cancer [170].

### Lung cancer

The complex of TF and VIIa, as well as the binding of epidermal growth factor to its receptor in cancer cells present in lung cancer, leads to the activation of the mTOR pathway, and the mTOR pathway leads to an increase in the expression of TF in lung cancer and glioblastoma [73]. The Heparanase gene (HPSE) is expressed in physiological conditions by cells involved in the immune system, platelets, and placental cells. Heparanase leads to the breaking of the heparin sulfate chain in the extracellular matrix and cell surface. Recent studies showed that heparanase gene expression is controlled by p53 and EGR1. On the other hand, it has been shown that heparanase plays a role in lung cancer tumorigenesis and metastasis due to TF. But Sandra et al. showed that heparanase is not related to TF expression. The Kaplan–Meier diagram shows that patients with NSCLC with a high level of asTF have shorter survival than patients with a low level of TF [171]. Therefore, asTF could be a valuable prognostic marker in NSCLC. Examination of NSCLC shows that the increase in TF leads to an increase in angiogenesis through the increase in VEGF expression [172]. Over-expression of TF in murine tumor cells increases VEGF production while decreasing thrombospondin production, and under-expression of TF decreases VEGF production while increasing thrombospondin production [173]. GOLDIN-LANG et al. quantify mRNA and protein levels of flHTF (the physiological initiator of blood coagulation) and asHTF (a soluble isoform of TF) in human NSCLC tissue and specimens collected from plasma. To reach this target, they evaluated the expression of TF in 21 pulmonary adenomatous (AC) and 12 normal healthy tissues by real-time qRT-PCR. The fold change was 3.4 i.e., pulmonary adenomatous mRNA expression was 3.4-fold higher than control specimens. Another report realized from this study is that the expression of AsHTF mRNA was markedly lower in patients with stage IA disease than patients with higher grade stages, indicating TF could be a marker of malignancy and metastases. Finally, this study revealed that the higher expression of flHTF and, especially, asHTF in AC, is associated with an increased risk of thrombosis, metastasis, and tumor progression so, indicating a poor prognosis in these patients [3]**.**

The level of TF is increased in advanced stages as well as 3 or 4 grades of NSCLC. The TF level expression and VEGF-189 are increased in NSCLC lung cancer that has a mutation in codon 12 of the *KRAS* gene. Therefore, this study revealed that, in general, the level of TF, microvascular density (MVD), angiogenesis, and VEGF-189 in lung cancer is reduced, but if the lung cancer progresses to advanced stages, such as NSCLC, and If cancer cells have a point mutation in the Ras gene, the expression of the abovementioned factors could be increased [174]. The study of J.ROLLIN revealed that TF gene expression has a strong relationship with the VEGF-189 [171]. When compared to cancers with strong TF expression, TF-negative lung malignancies were more commonly resistant to doxorubicin. Furthermore, Koomägi et al. discovered that patients with lung carcinomas that were TF-positive had lower survival periods than individuals whose cancers were TF-negative [146]. Therefore, TF may have prognostic significance and be used as a marker to assess the likelihood of survival in NSCLC.

### Hepatocellular carcinoma

Hepatocellular carcinoma (HCC) is a malignant tumor that has a high propensity for invasion and metastasis [175]. One of the important factors that lead to the aggressiveness and malignancy of this carcinoma is VEGF-induced angiogenesis, which increases following the increase in TF expression [176–178]. The assessment of angiogenesis through the measurement of microvessel density is followed by the assessment of the CD34 marker as an endothelial marker. Therefore, by targeting VEGF and TF, HCC growth and metastasis can be prevented [140]. Poon et al. study: TF expression in HCC relates to microvessel density, VEGF level, metastasis, survival rate, and poor prognosis; also, serum VEGF is a prognostic factor in HCC [140].

Poon et al. study also reveals that there was no highly significant association between tumor TF levels and tumor size. Tumor TF expression may affect tumor invasiveness regardless of tumor size. Also, in their research, Patients who had a high tumor TF level had a considerably worse prognosis than patients who had a low tumor TF level [140]. This finding provides evidence that TF plays a significant part in the aggressiveness of HCC. In addition to its prognostic value, the association of TF with angiogenesis and tumor invasiveness may have therapeutic implications [140]. Because of the vascularity of HCC, antiangiogenic therapy has a great deal of potential for treatment [179]. Antiangiogenic therapy could be beneficial as adjuvant therapy in individuals undergoing HCC resection. HCC growth has been proven to be inhibited by VEGF therapy [180]. In HCC, TF may be a novel target for antiangiogenic therapy. In animal models, blocking TF activity using monoclonal antibodies inhibited tumor metastasis [181]. Pentoxyphylline [182] and retinoic acid can similarly inhibit tumor TF expression [183].

### Cervical cancer

One of the factors that play a role in the progression of metastasis and invasion of this disease is the expression of TF. Studies show that tumor cells, by activating the coagulation system, eventually lead to an increase in blood viscosity and a decrease in blood speed, which leads to thrombosis and invasion of tumor cells. In this cancer, as the disease progresses, the expression of TF increases, which indicates the relationship between TF and the degree of the disease. So Tissue factor could be determined as a novel marker for the detection of cancer cells circulating in the body [65, 184]. Bono et al. conducted a study in which immunohistochemistry was used for tissue factor expression. Patient-match tumor biopsies were stained via TF-specific antibodies in the immunohistochemistry method. They observed that the highest prevalence of the expression of tissue factor belonged to cervical cancer and some other cancer like glioblastoma, pancreatic cancer, colon cancer, and NSCLC [185].

### Retinoblastoma

This tumor is one of the tumors that have a lot of blood vessels and affected patients have a high risk of thrombosis, which worsens cancer. Various growth factors play a role in the progression, growth and drug resistance of this tumor. Among all these growth factors, the main growth factor that plays a role in the development of retinoblastoma is FGF-2. In fact, FGF-2 in the tumor environment leads to increased angiogenesis [43]. A study conducted by Song et al. demonstrated that TF regulates this angiogenesis in retinoblastoma tumors. In the proliferative region of retinoblastoma, which includes tumor cells as well as tumor vessels, TF was preferentially expressed. This expression co-localized with tumor vessel endothelial cells [43]. This could imply the TF's role in progressing cancer.

### Gliomas

Hamada et al.[127] discovered that only one benign glioma out of ten (10%) was found positive for TF (grade I–II), whereas 19 of 20 glioblastomas (95%) and 13 of 14 anaplastic astrocytomas (86%) tested moderately or highly positive for TF. Moreover, Guan et al. [126] findings supported those of Hamada et al. had reported. Overexpression of TF in mouse sarcoma cells enhances angiogenesis by increasing the synthesis of VEGF, a positive angiogenic factor, and decreasing the production of thrombospondin 2, a negative angiogenic factor [173]. Guan et al. reported that there is a significant correlation between TF expression and tumor microvessel density, a measure of angiogenesis, which suggests that TF may play a role in glioma angiogenesis induction. As a result, they noted that TF is highly expressed in gliomas and that the degree of expression is correlated with the microvascular density and histologic grade of malignancy [126].

Several cancers cause abnormal blood clotting, which is characterized by deep vein thrombi (DVT) and pulmonary emboli (PE), collectively known as venous thromboembolism (VTE). One of the cancers that is susceptible to VTE is gliomas. Noteworthy that isocitrate dehydrogenase 1 (IDH1) mutation status is one of the most effective prognostic markers for VTE in glioma [186]. Gliomas can be divided into two general categories with point mutations and without point mutations. Approximately 20–30% of point mutations in glioblastomas occur in IDH1 and, to a lesser extent in IDH2, which are enzymes involved in cellular metabolism. One of the most common genes with hypermethylation and reduced expression in mutant IDH glioma is F3 (TF coding gene). This hypermethylation is due to the production of 2-hydroxyglutarate (2-HG) caused by the mutation in IDH, which changes the expression of many genes. Mutant type IDH1 has a lower risk of VTE compared to the wild type, and the amount of TF in it is also lower. Mutant type IDH1 is less invasive than its wild type due to hypermethylation of the TF gene and reduced expression. Therefore, demethylating compounds such as DAC (deacetylase) have adverse effects in mutant IDH1. But the use of DAC in wild-type IDH1 reduces cell proliferation [31]. So, two possible complementary pathways are known for how mutant IDH1/2 prevents thrombosis. The first is methylation-based; mutant IDH1/2 causes F3 promoter hypermethylation, resulting in decreased transcription of the F3 mRNA and reduced production of the TF protein [187]. Mutant IDH1/2 promotes gliomagenesis and inhibits GBM growth by inhibiting intratumoral thrombi development and necrosis. This explains why most mutant IDH1/2 gliomas are not grade IV, while most wild-type gliomas are [186]. IDH1 mutant gliomas without intratumoral microthrombi have the best prognosis, regardless of patient age. In wild-type IDH1 gliomas, increased TF expression and the presence of microthrombi correlate with a 50% decrease in survival. TF expression enhances migration, adhesion, and proliferation in both IDH1 mutant and wild-type gliomas. High levels of D-2-HG, exceeding 100 M, are found in the cerebrospinal fluid of mutant IDH1/2 glioma patients, suggesting significant concentrations in the tumor microenvironment. D-2-HG's antiplatelet activity cannot be attributed to methylation due to its immediate action and platelets lacking DNA [186, 188].

A study revealed that suppression of TF inhibited the growth of GBM12 in vivo but not GBM6. Although GBM43 did not express EGFR, it did express PDGFRβ, which is an expressed type of RTKs activated by TF. TF is dependent on RTKs (receptor tyrosine kinase) for inducing cell growth and proliferation and tumorigenesis [31]. One of the important factors that is located downstream of TF and leads to the activities mentioned above by TF, is beta-catenin. Activation of Par2 leads to stabilization of beta-catenin and beta-catenin itself leads to cell-to-cell and cell-to-matrix adhesion during tumor cell invasion. Therefore, the important point is that for the growth of tumor cells, we need the activity of TF and RTKs, and for the invasion of tumor cells, we need the relationship between TF and beta-catenin. TCGA gene analysis shows a positive relationship between ECM-receptor interaction with mRNA, F3 in glioma [31].

Disruption of the blood–brain barrier in glioblastoma leads to an increase in TF levels in the blood [125]. Nobuhiro et al. revealed that increased expression of TF is related to lymph node metastasis and the degree of cancer malignancy, so patients whose tumor cells do not express TF have a better prognosis than those whose tumor cells do, even if there is lymph node metastasis [62]. In the tumor microenvironment, there are two types of macrophages: M1 and M2. M1 macrophages promote tumor destruction and produce TNF and interleukin-6, while M2 macrophages contribute to tumor progression by producing VEGF, MMP, and interleukin-10. M2 macrophages are particularly involved in tumor angiogenesis. The presence of TAMs (tumor-associated macrophages) decreases in late-stage TFδCT and TFδCT/par2-/- tumors. Alternative splicing of VEGF mRNA generates four isoforms (121, 165, 189, and 206 amino acids), each with specific functions. TF, through PAR1/2 pathways, regulates VEGF expression, leading to angiogenesis and invasion in various cancers. The larger isoforms (189 and 206 amino acids) are bound to glycosaminoglycans, while the smaller isoforms (121 and 165 amino acids) are secreted into the extracellular matrix. Both 189 and 165 amino acid isoforms contribute to angiogenesis, while only VEGF189 is involved in cell growth [174].

## TF targeted and re-targeted therapy

### TF targeted therapies and targeted diagnostic approaches

TF is highly expressed in different kinds of cancers. It activates the extrinsic blood coagulation pathway and enhances tumor progression and metastasis [189, 190]. Many studies have shed light on the association between blood coagulation and cancer progression [191, 192]; Therefore, TF-targeted therapies can be effective in reducing tumor growth, angiogenesis, and metastasis in many cancers (Table 3) [59, 60, 189, 190]. Not only is targeting TF considered for cancer treatment, but it also helps for the precise detection of TF expression in tumors.

**Table 3:** Targeting  TF for cancer therapy

Agent	Target	Model	Outcome	Ref
**Monoclonal antibody**				
Anti-β1 integrin antibody (HUTS-21)	β-tail domain (βTD) of the β1 integrin subunit	Breast cancer models in vitro and in vivo	Reduction of the asTF-dependent proliferation of tumor cells	[60]
Anti-asTF antibody (Rabmab1)	asTF	PDAC cell line (Pt45.P1) in vivo	progression and spread restriction	[59]
Anti-TF antibody SC1	TF extracellular domain, PAR2 signaling	Broad triple-negative breast cancer cell lines and Pancreatic adenocarcinoma cancer cell lines in vitro and in vivo	inhibition of TF-induced cell migration, lung metastasis, and tumor growth. Diminished levels of tumor angiogenesis and stromal fibrosis of triple-negative breast cancer and Pancreatic adenocarcinoma cancer	[190]
10H10	TF (signaling)	Murine model In vivo	Delayed progression of GBM cells harboring EGFRvIII	[193]
CNTO 859	TF (coagulation)	Murine model In vivo	Delayed progression of GBM cells harboring EGFRvIII	[193]
**Antibody drug conjugate**				
SC1-DM	TF	Broad TNBC and PaC cell lines in vitro and in vivo	Cytotoxic effects in TF-positive TNBC and PaC cancers	[190]
SC1-MMAE	TF	Broad TNBC and PaC cell lines in vitro and in vivo	Cytotoxic effects in TF-positive TNBC and PaC cancers	[190]
Tisotumab vedotin (InnovaTV 201)	TF	Broad cancers, clinical trial	Anti-cancer activity	[194–197]
Anti-human TF (clone 1849)-MMAE	TF	several pancreatic cancer cell lines, in vitro and in vivo	Suppression of tumor growth	[198]
Anti-TF1859-NC-6300 (epirubicin-incorporating micell)	TF	BxPC3 and SUIT2 pancreatic cancer, in vitro and in vivo	Anti-cancer activity	[199]
Anti-TF-NC-6300	TF	BxPC3, SUIT2 and 44AS3 pancreatic and gastric cancer in vitro and in vivo	Anti-cancer activity	[200]
TF-011-MMAE	TF interruption of TF: FVIIa-dependent intracellular signaling	patient-derived xenograft (PDX) originating from seven diverse solid cancers in vitro and in vivo	Anti-cancer activity	[189]
mfVII/human Fc icon	TF/chondroitin sulfate	Several cancer models	Activation of the complement system and NK cell inhibition of growth and relapse of an established human tumor model	[201–206]
PAR1 pepducins	first (i1) and third (i3) intracellular PAR1 loops	lung cancer cell lines in vivo	meaningful cell migration hindrance	[207]
PAR1 pepducin	third (i3) intracellular PAR1 loop	lung cancer cell lines in vivo	Inhibition of cancer cell proliferation	[207]
imidazopyridazine compound I-191	PAR2	MDA-MB-231 human breast adenocarcinoma cell line and HT29 human colon adenocarcinoma cell line	Inhibition of PAR2 intracellular signal pathways	[208]
**TF ligand inhibitor**				
Rivaroxaban	coagulation factor X (FX) and PAR2 signal transduction	Peripheral blood monocytes and neutrophils and murine model In vitro, in vivo pancreatic tumor models In vivo	increasing infiltration of dendritic cells and cytotoxic T cells at the tumor region, but could not reduce growth of pancreatic tumor models	[209, 210]
PCI-27483	TF: FVIIa-complex and PAR2 signaling	BXPC3 pancreatic tumor mice model	significant dose-dependent tumor growth	[211]
rNAPc2				
rNAPc2	TF/FVIIa complex inhibition	B16F10 melanoma cell line In vivo	primary and metastatic tumor growth and angiogenesis	[212]
CAR cell				
TF-CAR T cell	TF	non-small cell lung cancer (NSCLC) cells and melanoma cells in vitro and in xenograft and metastasis models of human NSCLC in NOG mice	In vitro: strong cytotoxic potential In vivo: significant suppression of s.c. xenograft growth and lung metastasis models	[213]
TF-CAR NK cell (single and combination therapy with L-ICON)	TF	TNBC cell lines in vitro TNBC cell lines and patient’s tumor-derived xenograft mouse models in vivo	in vitro: TF-CAR-NK cells alone could kill TNBC cells, and its efficacy was enhanced with L-ICON ADCC in vivo: effective treatment of TNBC cell line- and patient’s tumor-derived xenograft mouse models	[27]
**Vitamin K antagonist**				
Warfarin	Gas6-dependent Axl activation	Pancreatic cancer cell lines in vitro and in vivo	Inhibition of development, spread, migration, invasiveness, and proliferation of pancreatic cancer cells	[214]
**Antibody-mediated imaging**				
Anti- TF antibody (ALT-836)	factor X/factor IX (FX/FIX) binding site of TF	surgical endarterectomy model in chimpanzees In vivo	Prevention of thrombin generation and acute vascular thrombosis	[215]
64Cu-NOTA-ALT-836	factor X/factor IX (FX/FIX) binding site of TF	Pancreatic cancer cell lines in vitro, in vivo and ex vivo Thyroid cancer cell lines In vivo	More uptake in BXPC-3 tumors (high TF expression) than in PANC-1 and ASPC-1 tumors (lower TF expression Suppression of subcutaneous and orthotopic anaplastic thyroid cancer (ATC) with high levels of tumor uptake	[216, 217]
(64)Cu-NOTA-ALT-836-Fab	factor X/factor IX (FX/FIX) binding site of TF	MDA-MB-231 TNBC model	Several-fold greater tumor uptake compared to the blocking group and tumor models that failed to significantly express TF	[218]
^89^Zr-Df-ALT-836	factor X/factor IX (FX/FIX) binding site of TF	Pancreatic cancer cell lines in vitro, in vivo, ex vivo	long-term and prominent uptake in BXPC-3 tumors	[219]
Heterodimer of TRC-105 and ALT-836 dual radiolabeled with 64Cu heterodimer-ZW800	TRC-105: CD105 ALT-836: factor X/factor IX (FX/FIX) binding site of TF	Mice models of pancreatic cancer	High tumor uptake	[220]
^64^Cu-NOTA-ALT-836 IRDye 800CW-ALT-836 (near-infrared fluorescent imaging probe) ^131^I-ALT-836 (radioimmunotherapy)	factor X/factor IX (FX/FIX) binding site of TF	Anaplastic thyroid cancer (ATC) in vivo and ex vivo	a peak tumor uptake leads to both subcutaneous and orthotopic suppression of ATC prolongs the survival of ATC-bearing mice	[217]
18F-FVIIai	TF	mouse model of human pancreatic cancer In vivo and ex vivo	significant uptake of 18F-FVIIai by TF-expressed tumors	[221]

#### Antibody

Various types of antibodies have been used in studies, including anti-asTF monoclonal antibodies, antibody drug-conjugate (ADC), and molecule-conjugated Fc of IgG1. AsTF is a secreted form of TF that induces blood coagulation in patients with heightened TF expression levels in many forms of solid tumors, such as pancreatic ductal adenocarcinoma (PDAC). Also, RabMab1, as an asTF-specific neutralizing antibody, is capable of restricting PDAC progression and spread [59].

Many studies have assessed the anti-tumor effects of ADC on a broad range of solid tumors [189, 190, 194–200, 222–224]. In this approach, the antibodies must bind to a tumor-specific antigen that is highly expressed on the cell surface that can be internalized into the tumor cells by an endosomal vesicle. The drugs conjugated to antibodies must be high-potential cytotoxic agents, and the linkers must be persistent in the physiological situation, such as plasma, and cleaved properly by proteases like cathepsin B in an acidic environment of cell lysosome [225, 226]. A specific anti-TF antibody, called SC1, demonstrates a remarkable efficacy against TF extracellular domain, intracellular PAR2 signaling, and tumor-initiated coagulation, which is efficient against TF-positive triple-negative breast cancer (TNBC) and Pancreatic adenocarcinoma cancer (PaC) cells. Accordingly, depleting the TF or SC1-treatment in TNBC or PaC cells led to the inhibition of TF-mediated cell migration, lung metastasis, and tumor growth. Besides, tumor capability for angiogenesis and stromal fibrosis was reported to be reduced. SC1-DM1(emtansine) and SC1-MMAE (monomethyl auristatin E) developed due to TF's quick and effective internalization of SC1-drug conjugate. Both of them induced excellent cytotoxicity in TF-positive TNBC and PaC cells [190].

A first-in-human antibody–drug conjugate is Tisotumab vedotin (InnovaTV 201), which targets TF directly and is in phase I/II of a clinical trial. It consists of a fully human monoclonal antibody that specifically binds to TF, conjugated to the MMAE via a protease-cleavable linker. Tisotumab vedotin is a potent TF-ADC that has promising anti-cancer activity in severely pretreated patients with several distinct cancers known to express TF [194]. As reported for the cervical cancer cohort of the innovaTV 201 phase I/II study (NCT02001623) and phase II clinical trial study, Tisotumab vedotin exhibited a controllable and tolerable safety profile along with significant and prolonged anti-cancer activity, In patients who were formerly treated recurrent or metastatic cervical cancer [195, 196]. A primary paratope family which did not affect the alteration of Factor X (FX) to activated Factor X (FXa) and had no effects on the alteration of prothrombin to thrombin is used. Both the coagulation-inert MMAE-anti-TF antibodies and inhibitory conjugated MMAE-anti-TF antibodies (tisotumab vedotin) killed tumor cells significantly [197]. MMAE-anti-human TF (clone 1849), capable of internalizing into cells, has been used on cell lines expressing TF, which was followed by efficient MMAE release and notable suppression of pancreatic tumor growth [198]. ADC anti-tumor potential depends on kinetics parameters. In a study, three types of anti-TF ADCs were established, consisting of a low k_d_ 1849ADC, an intermediate k_d_ 444ADC, and a high k_d_ 1084ADC. This study selected MMAE and the MC-vc-PABC linker, which is a broadly used and extremely potent anti-cancer combination [227]. *In the *in vivo, all ADCs exhibited the same anti-cancer effects against a small BxPC3 model of pancreatic tumor. While on a larger BxPC3 model of pancreatic tumors, 1084ADC (higher k_d_) indicated greater anti-cancer activity in comparison with 1849ADC (lower k_d_). Besides, comparing 1849ADC to 1084ADC via exerting immunofluorescence staining exhibits that the distribution of the latter is across the whole tumor, whereas the former only localizes nearby the tumor vessels. The anti-tumor effects are augmented by the ADC with a greater k_d_, due to its penetration and distribution across the whole solid tumor [222].

There is another ADC, called an anti-TF1849 antibody, with a potent anti-tumor activity although an inhibitory effect on the blood-coagulating activity of TF. In contrast, the anti-TF1859 has the drawback of anti-TF1849, which led to the development of three other forms, namely anti-TF1859-IgG-NC-6300, anti-TF1859 F (ab’)2-NC-6300, and anti-TF1859-Fab’-NC-6300. The NC-6300 is an epirubicin-incorporating micelle that has an extensive anti-tumor effect [228]. Anti-TF1859-IgG-NC-6300 has greater anti-cancer activity in cells with highly expressed TF [199]. In another study, anti-TF-NC-6300 having the F (ab')2 of anti-TF mAb was established to be compared to NC-6300 both in vitro and in vivo*.* This particular ADC was tested on BxPC3 (high TF expressing pancreatic cell line), 44As3 (high TF expressing gastric cell line), and SUIT2 (low TF expressing pancreatic cell line) xenografts. Anti-TF-NC-6300 showcased a greater anti-cancer capability in BxPC3 and 44As3 xenografts, whereas both agents indicated similar activity in the SUIT2 model. Anti-TF-NC-6300 seemed capable of localizing to the high TF-expressing cancer cells. Results showed that the heightened anti-cancer effect of anti-TF-NC6300 might rely on the selective intratumor localization and the preferential internalization of this ADC into high TF-expressing cancerous cells [200].

Conjugated anti-TF mAb (a chimeric antibody composed of clone 1849 variable domains and constant human domains) to immunomicelles exhibits higher anti-cancer potential toward TF-positive stomach cancer compared to the anti-TF mAb and NC-6300 combination; it can be distributed more equally among TF-positive tumor tissue in comparison with NC-6300. On the other hand, anti-HER2 mAb (trastuzumab) conjugated to immunomicelles does not indicate meaningful anti-cancer activity toward HER2-positive stomach cancer in comparison with the combined use of anti-HER2 mAb and NC-6300 in gastric cancer [223]. Moreover, a study compared TF-ADC to EGF-R family receptors, TF, HER2 and EGF-R specific monoclonal antibodies conjugated to duostatin-3 (an antimitotic agent which hinders tubulin) polymerization). TF-011, HER-2 005, and zalutumumab are Monoclonal antibodies that target TF, HER2, and EGF-R. The study showed that TF-ADC is more efficient than the EGF-receptor family, whereas lower expression of TF compared to the expression of HER2 or EGFR is reported. It is hypothesized that to have an appropriate ADC treatment approach, the perpetual TF turnover on tumor cells is needed [224].

Another ADC called TF-011-MMAE, a compound of human TF-specific mAb conjugated to MMAE by a protease-cleavable linker, is able to interrupt TF:FVIIa-dependent intracellular signaling and eliminates cancer cells in vivo without affecting TF procoagulant activity in many solid tumors. TF-011-MMAE showcased remarkable anti-cancer effects in the preclinical stage on patient-derived xenograft (PDX) models with different TF expression levels originating from seven distinct solid tumors. Analyzing both the antibody and MMAE parts of this ADC showcased that the auristatin part of MMAE mediated most cytotoxic effects on cancer cells [189].

Molecule-conjugated Fc of IgG1 is an effective approach for immunotherapy. The natural killer cell (NK cell) is a prominent cell of the innate immune system, which can be a potent cytotoxic for a cancer cell; if it is stimulated. The complement system, as an important part of the immune response, can interfere with cancer cells in the presence of antibodies. The classic pathway of the complement system is activated when at least two Fc domains bind to C1q simultaneously. The reason for using IgG1 is its potential for complement fixation. By conjugation of the tumor-targeting domain with the human IgG1 Fc effector domain and interaction with FC receptors like CD16a, NK cell can be activated and has a cytotoxicity effect by a mechanism named antibody-dependent cellular cytotoxicity (ADCC) [201]. Two kinds of targeting domains were studied in a severe combined immunodeficient (SCID) murine model of human melanoma and prostatic cancer. Intratumoral injections of the adenoviral vector encoding the mfVII/human Fc icon into the skin tumor, led to the synthesis of the icon by tumor cell. When the icon binds to TF expressed on cellular membrane of endothelial cells that line the tumor vasculature lumen and also to human TF expressed on cellular membrane of tumor cells, it can activate the complement system and NK cells against them [202]. Two targeting domains are the human single-chain Fv molecule and factor VII (fVII). The former interacts with a proteoglycan named chondroitin sulfate that is mostly expressed on the cellular membrane of human melanoma cells. The latter is a zymogen that is capable of high-affinity interaction with TF to trigger the blood coagulation cascade. Several kinds of tumor cells and also endothelial cells that line the tumor vasculature express TF, whereas the normal vasculature does not. In the study, due to the interaction of an fVII immunoconjugate to TF that may lead to dispersed intravascular clotting, the fVII active site mutation was done to hinder clotting without having an effect on the affinity for TF. The results indicated the inhibition of growth and relapse of an established human tumor model. This approach showed the capability of efficiency for a wide range of solid tumors treatment [201–205, 229]. A noticeable point is the importance of NK cells in this treatment, and studies showed that impaired NK activity or level could lead to resistance to immunotherapy by antibodies [201].

#### PARs antagonist

Protease-activated receptors (PARs) are specific GPCRs, large superfamily members which trigger cellular signaling as a response to extracellular proteases. Four members of the PAR family have been identified. PAR1 is the main thrombin receptor that acts as an encouraging target to affect cancer progression, metastasis, and angiogenesis in many cancers, including colon, breast, prostate, melanoma, and ovarian cancer [230–233]. Besides, PAR1 is the only one that was significantly expressed in the cell lines of lung cancer [207]. When PAR1 is cleaved by thrombin, it is activated. The cleavage occurs between the N-terminal extracellular domain residues R41 and S42 of the receptor. [234]. Various proteases can activate PAR1 coupled with Gα-subunits. All three heterotrimeric Gα-subunits can be activated simultaneously by thrombin [235]. Cell-penetrating pepducins inhibitors are specific for the first (i1) and third (i3) intracellular PAR1 loops. The PAR1 pepducins exhibited meaningful cell migration hindrance in lung cancer cell lines, the same as PAR1 expression silencing with short hairpin RNA (shRNA). The i3 loop pepducins but not i1, were profound inhibitors of PAR1-mediated ERK activation and also tumor cell proliferation [207]. PAR2 is a trypsin/tryptase receptor that is highly expressed in cancer cells [207, 236, 237]. A study identifies imidazopyridazine compound I-191, as an antagonist for PAR2 activation. Interaction of I-191 with PAR2 cause a noncompetitive modulation and acts as a negative allosteric modulator of the agonist 2f-LIGRL-NH_2_. Therefore, Various intracellular signal transduction pathways followed by PAR2 are effectively debilitated. Inhibition of PAR2 activation by I-191, cause a remarkable inhibition in all PAR2-dependent intracellular signal transduction pathways such as Ca^2+^ release, phosphorylation of extracellular signal-regulated kinase 1/2 (ERK1/2), Ras homolog gene family, RhoA activation, and forskolin-induced cAMP accumulation. Also, I-191 significantly hindered PAR2-mediated downstream functional responses, such as inflammatory cytokines expression and secretion, cellular apoptosis, and migration in MDA-MB-231 cell line (human breast adenocarcinoma) and HT29 cell line (human colon adenocarcinoma) [208].

#### TF ligand inhibitor

Cancer cells expressing TF, platelets activation, and interaction between platelet and leukocyte paves the way for cancer cell survival in the blood and metastasis, Whereas TF-FVIIa interaction activates PAR2, which improves cancer progression without the dependency on the intravascular blood coagulation cascade. Tumor-associated macrophages (TAM) and monocytes produce the FVII and FX, which can activate PAR2. FXa-PAR2 signaling hampers anti-cancer immune response in the TME and leads to immune evasion. Rivaroxaban; is a small molecule that inhibits coagulation factor X (FX) activation and enhances anti-cancer immune response by increasing infiltration of dendritic cells and cytotoxic T cells at the tumor region. It targets PAR2 signal transduction specifically to reprogram TAM [209]. The other stud used rivaroxaban as an inhibitor of Factor Xa in both BxPc-3 and MIA PaCa-2. Results showcased high TF expression in BxPc-3 while no TF expression in MIA PaCa-2 is reported. As a result, the growth of TF-positive BxPc-3 tumors can be reduced by rivaroxaban but not by TF-negative MIA PaCa-2 tumors in nude mice. Also rivaroxaban has no effect in the proliferation of both breast cell lines [210].

PCI-27483 is a small molecule that inhibits the TF: FVIIa-complex and signaling downstream of PAR2, including MAPKs and Akt phosphorylation, c-fos induction, vascular endothelial growth factor (VEGF), and IL8 secretion as an autocrine growth factor known to induces chemotaxis and invasion in BxPC3 cell line (pancreatic adenocarcinoma). Besides, a significant dose-dependent tumor growth inhibition (in vivo) in mice implanted with BxPC3 cells is reported [211]. In the phase II clinical trial study, a combination of PCI-27483 with Gemcitabine was well tolerated, but it was insignificant in contrast to Gemcitabine [238].

#### rNAPc2 inhibitor

Recombinant nematode anticoagulant protein c2 (rNAPc2) is a small molecule that hampers TF/FVIIa complex and has antithrombotic effects [239]. It impedes complex enzymatic initiation in the blood clot cascade [240, 241]. A direct positive correlation between TF expression and vascular density and vascular endothelial growth factor (VEGF) expression is identified [242]. When rNAPc2 is used simultaneously beside 5-fluorouracil as a cytotoxic agent or bevacizumab (a humanized anti-VEGF monoclonal antibody) in mice with xenografted, disseminated, or spontaneous colorectal cancer cells, it inhibits primary and metastatic tumors growth [239]. rNAPc2 hampers both primary and metastatic tumor growth and is also an efficient angiogenesis inhibitor in mice [212]. A clinical trial study used combination therapy of rNAPc2 beside aspirin, clopidogrel, and unfractionated heparin and indicated that dose-dependent Impediment of the TF/FVIIa complex formation is safe and a profound approach to prohibit thrombin production through coronary angioplasty [243]. The higher dose of rNAPc2 inhibited prothrombin fragment F1.2 generation and also decreased ischemia by more than 50% in comparison with the placebo and rNAPc2 lower dose [244].

#### CAR cell-mediated immunotherapy

Chimeric antigen receptor (CAR) is a novel genetically modified super receptor that is expressed on T and NK cells and can bind specifically to cancer cell antigens. Therefore, CAR-T and CAR-NK cells are known as promising immunotherapeutic approaches for cancer treatment [27, 213, 245, 246]. Clinical trial studies have illustrated that CAR-T cell transferring to patients suffering from cancer provides a novel strategy to transport specific antigen-targeted cancer treatment [247, 248]. Zhang and colleagues designed a new third-generation CAR that targets TF (TF-CAR T). In their study, mouse FVII (mlFVII) is selected as the TF-CAR target-binding domain. To determine the capability of TF-CAR T cells, in vitro and in vivo studies were carried out. The former indicated a strong cytotoxic potential of TF-CAR T cells against TF-positive cancer cells, and also, the latter showcased a significant suppression of s.c. xenograft growth and lung metastasis models [213, 249]. Additionally, CAR-NK cells were evaluated in triple-negative breast cancer (TNBC). The absence of a targetable surface molecule has made it an incurable cancer. In a study, TF-CAR-NK cells co-expressing CD16 (FcγRIII) were designed to mediate antibody-dependent cellular cytotoxicity (ADCC). The study developed a second-generation TF-targeting antibody-like immunoconjugate (named L-ICON). In preclinical evaluation, a comparison of both TF-CAR-NK cells (single-agent therapy) and TF-CAR-NK cells with L-ICON (combination therapy) was conducted. As a result, TF-CAR-NK cells were capable of killing TNBC cells, and as the combination therapy, its effectiveness was boosted with L-ICON ADCC in vitro. Additionally, TF-CAR-NK cells were successful in treating TNBC in vivo using patient's tumor and cell line-derived xenograft mice models [27, 250–258].

#### Anticoagulant

Long-term consumption of vitamin K antagonists like warfarin can reduce the incidence of cancers. In a study that supports the hypothesis that anticoagulation may affect protectively cancer development, vitamin K antagonist-exposed patients were lower likely to develop prostate cancer in comparison with the control group [259]. Besides, the risk of patients suffering from cancer recently after the first episode of venous thromboembolism appears to be minor among patients treated with oral anticoagulants like warfarin for a six months period [260]. As a molecular mechanism of action for warfarin, a study reported that hindering Gas6-dependent Axl activation with warfarin inhibits the development, spread, migration, invasiveness, and proliferation of pancreatic cancer. However, it enhances apoptosis and susceptibility to chemotherapy [214].

#### Antibody-mediated imaging

Positron emission tomography (PET) imaging of TF is a profound diagnostic technique applicable for tumor staging in a wide variety of malignancies [216]. ALT-836, a recombinant IgG4κ chimeric antibody that blocks the factor X/factor IX (FX/FIX) binding site of TF, showed extreme efficiency at preventing thrombin generation. In a chimpanzee arterial thrombosis model caused by surgical endarterectomy, it reduced acute vascular thrombosis with no discernible differences in surgical blood loss and template-bleeding time in the treated group compared to control animals [215]. In the phase I clinical trial study, it was demonstrated that in patients with ALI/ARDS caused by sepsis, dose-dependent ALT-836 could be safely administered without having an anti-ALT-836 antibody response and major bleeding periods. The most common side effects were anemia [261]. NOTA-ALT-836, a 64Cu (radiolabeled) conjugate of ALT-836 and 2-S-(4-isothiocyanatobenzyl)-1, 4, 7-triazacyclononane-1, 4, 7-triacetic acid (p-SCN-Bn-NOTA), is utilized for immuno-PET imaging [216, 262]. In vivo PET imaging of mice with pancreatic cancers revealed more 64Cu-NOTA-ALT-836 uptake in BXPC-3 tumors (high TF expression) than in PANC-1 and ASPC-1 tumors (lower TF expression) [216]. Besides, it delimits both subcutaneous and orthotopic anaplastic thyroid cancer (ATC) with high levels of tumor uptake [217]. A near-infrared fluorescent imaging probe (IRDye 800CW-ALT-836) paves the way for the entire resection of orthotopic ATCs and ^131^I-ALT-836 as a therapeutic conjugate for radioimmunotherapy (RIT), enhances the survival rate of ATC-bearing mice [217].

Long-lasting ^89^Zr-Df-ALT-836 PET scans revealed a long-term and prominent uptake in BXPC-3 tumors while pre-injection of mice with a blocking dose of unlabeled ALT-836. It indicates the excellent affinity and TF-specificity of this radiolabeled PET-tracer [219]. Several-fold greater tumor uptake of (64) Cu-NOTA-ALT-836-Fab compared to the blocking group and tumor models that failed to significantly express TF was seen in a serial PET imaging study of the MDA-MB-231 TNBC model [218]. Dual-targeted imaging agents have indicated enhanced targeting effectiveness rather than single-targeted entities. A heterodimer from the Fab′ fragments of TRC105 (IgG_1κ_ monoclonal antibody targeting CD105) and ALT-836 (IgG_4_ monoclonal antibody targeting TF) is designed. Heterodimer was radiolabeled with 64Cu before being injected into mice models of pancreatic cancer. PET scans using 64Cu-NOTA In contrast to Fab fragment homodimer, heterodimer-ZW800 in BxPC-3 tumor xenografts showed considerably higher tumor uptake [220]. A specific and non-invasive PET tracer called 18F-FVIIai uses active site-inhibited factor VIIa that has been radiolabeled with 18F. Four hours after injection, TF-expressed tumors showed significant uptake of 18F-FVIIai, and there is a link between this uptake and TF expression as evaluated ex vivo in tumor homogenates [221]. Comparing 1849-Fab-AF647 to 1849-whole IgG-AF647, the former demonstrated rapid dissociation from TF, tumor accumulation, tumor penetration, and also rapid body clearance. Thus this is an appropriate diagnostic tool [263].

### Re-targeted TF-based therapies

Occluding tumor blood supply is an appealing approach for treating tumors [264]. Vascular targeting therapy includes two strategies currently: inhibition of new vessel formation by antiangiogenic agents and elimination of existing tumor vasculature. The last one leads to the obstruction of pre-existing tumor blood vessels [265–268]. Targeted delivery of the extracellular domain of TF (truncated TF, so-called tTF) as a protein capable of coagulation induction to tumor vessels has been extensively investigated to achieve this goal. Free tTF is soluble and disabled to activate the blood coagulation pathway; however, its ability to induce clot is regained when localized close to a phospholipid membrane of cells. tTF activates blood coagulation pathways via activation factor X. Blood coagulation pathways (Both intrinsic and extrinsic) lead to the activation of clotting factor X. As a result, the conversion of prothrombin to thrombin occurs by activated factor X, which finally accumulates fibrin polymers, forms the polymeric fibrin network, and blood clots (Table 4).[13, 22–26]

**Table 4:** Re-targeted TF for cancer therapy

Agent	Target	Model	Outcome	Ref
**Antibody-mediated coagulation**				
Antibody-tTF (B21-2/10HlO-tTF) + IFN-γ	MHCII (IFN-γ-induced)	C1300(Muγ) mouse neuroblastoma	Thrombosis, complete tumor regression 38% of animals; volume reduction 70% Day 21	[269]
Anti.VCAM-1-tTF	VCAM-1	L540rec human Hodgkin’s lymphoma	Selective thrombosis; tumor inhibition but complete tumor regressions were not observed	[270]
Antibody-sTF (MK271 and 1G11B1) ± doxorubicin (DOX)	VCAM-1	L540rec human Hodgkin’s lymphoma Colo677 human small cell lung cancer (SCLC) ± human vasculature/HDMEC	Short term: tumor necrosis, 74% for L540rec + lipopolysaccharide, 26% for Colo677. Long-term: delayed tumor growth, 30% Colo677 (Day 14), with DOX 90%	[271]
chimeric antibody –tTF fusions (chTV-1-tTF)	Fibronectin	MAD109 murine lung carcinoma Colon-26 murine colon	Thrombosis and tumor growth inhibition	[272]
scFv-tTF	Fibronectin	F9 murine teratocarcinoma C51 murine colon carcinoma FE8 rat fibroblast sarcoma	High dose, complete tumor regression in 30%, rapid occlusion (1 h) in 50%	[273]
chimeric antibody –tTF fusions (chTNT-3-tTF)	Degenerated vasculature (exposed DNA)	MAD109 murine lung carcinoma Colon-26 murine colon	Thrombosis and tumor growth inhibition	[272]
scFV-tTF	TEM8	HT-29 human colorectal carcinoma	Localized thrombosis, 53% reduction in tumor volume	[274]
scFv- VIIa (TFOS4)	Fibroblast Activation Protein (FAP)	HT1080 cells	Induced plasma coagulation	[275]
**Peptide-mediated coagulation**				
RGD-tTF	α_v_β_3_ and α_v_β_5_	MAD109 murine lung carcinoma Colon-26 murine colon	Thrombosis, no significant inhibition of tumor growth	[272]
tTF-RGD (RGD coupled to the COOH-terminal of tTF)	α_v_β_3_ and α_v_β_5_	human adenocarcinomas (CCL185), melanoma (M21), and fibrosarcoma (HT1080)	Tumor growth retardation thrombotic occlusion of tumor vessels	[276]
RGD3-tTF (3 RGD repeat motifs)	α_v_β_3_	CT26 murine colorectal cancer	Thrombosis and necrosis, tumor growth inhibition, increased survival	[277]
tTF–truncated heparin-binding domain (HBD)	Complex (C6S, VEGFR2, NRP-1)	N202 murine mammary carcinoma	Rapid thrombosis, significant tumor eradication; FVIIa co-administration	[278]
SP5.2/tTF-OCMCs-SPIO-NPs	VEGFR-1	SMMC-7721 human hepatocarcinoma	Conjugated embolic nanoparticle thrombosis and vessel occlusion were observed > SP5.2-tTF alone	[279]
SP5.2-tTF	VEGFR-1	S180 murine sarcoma	Thrombosis, growth inhibition/regression 70% Day 6	[280]
tTF-EG3287	Neuropilin-1	HepG2 human liver cancer	Thrombosis and necrosis, tumor growth reduction	[281]
tTF-EG3287	Neuropilin-1	HT29 human colon adenocarcinoma	Thrombosis	[282]
tTF-EG3287 iron oxide NPs	Neuropilin-1	HepG2 human liver cancer	Thrombosis, necrosis, tumor growth inhibition	[283]
tTF-TAA tTF-NGR	NG2	A549 human lung adenocarcinoma M21 human melanoma	Lower anti-tumor activity and smaller therapeutic window of tTF-NGR relative to tTF-NGR	[284]
tTF-NGR	Aminopeptidase N (CD13)	A549 human lung adenocarcinoma M21 human melanoma HT1080 human fibrosarcoma + Low dosage clinical study (First-in-man, 5 terminal cancer patients	Animal studies: Vascular tumor volume reduction, regression at high s.c. but some toxicity	[285]
tTF-CREKA	Fibrin-fibronectin complexes	LS174T human liver cancer 4T1 human breast cancer MHCC97H human liver cance	Thrombosis, tumor growth inhibition	[286]
PSMA catalytic site inhibitor-tTF + Doxil	PSMA (prostate-specific marker antigen)	Rat Mat Lu prostate tumor LuCap human prostate tumor	Microvessel infarction, tumor growth inhibition	[287]
Antibody -streptavidin + tTF-biotin	Neuropilin-1	HepG2 human liver cancer	Thrombosis, tumor growth reduction and necrosis	[288]
CREKA-SPIO nanoparticles	Fibrin-fibronectin complexes	MDA-MB-435 human breast cancer	Low tumor growth inhibition	[289]
tTF-pHLIP	Acidic tumor matrix	MDA-MB-231 human breast cancer	Thrombosis, rapid tumor regression	[264]
tTF-pHLIP	Acidic tumor matrix	B16-F10 murine melanoma	Tumor growth inhibition	[290]
**Non-coagulant application**				
RGD-Doxorubicin NGR-Doxorubicin	α_v_β_3_ and α_v_β_5_ NG2 aminopeptidase N (CD13)	MDA-MB-435 human breast cancer	Effective antitumor activity, prolongation of survival	[291]
CREKA-Lipo-T	Fibrin-fibronectin complexes	4T1 mouse breast cancer	Inhibited tumor metastasis	[292]
TV.1-IL-2	basement membrane antigen	LS 174 T human colorectal cancer ME-180 human cervical carcinoma	Enhance uptake of monoclonal antibodies	[293]
CREKA-Tris (Gd-DOTA)3	Fibrin-fibronectin complexes	4T1 mouse breast cancer	Effectively visualize metastases containing micro-metastases	[294]

Identifying molecular targets that are highly expressed on tumor vascular endothelium and absent from normal tissues and organs is one of the basics for tumor vascular targeted therapy [288]. Numerous studies have indicated that targeting a specific antigen on tumor vessels brings about inhibition and retardation of tumor growth. It has been reported as a potential and appropriate strategy for tumor embolization therapy [271, 280, 285]. Antibodies and peptides with affinity against tumor antigens have been utilized for specific targeting of tTF to tumor vasculature [272]. Most of the specific ligands are produced by cloning systems to allow rapid generation of recombinant vascular targeting agents (VTAs) [295]. One promising approach is antibody-mediated coagulation induction in tumor nodules to cut off their blood supply. This concept was first reported in a murine model using neuroblastoma cells transfected with the IFN-y gene. Those cells released IFN-y, which increased the expression of MHC-II molecules on tumor endothelial cells. MHC-II are not present in normal endothelial cells in mice; however, they are expressed in APCs and some epithelial cells, so they are potential targets for antibodies that fuse to tTF. B21-2/10HlO bispecific antibody targets I-A^d^ and I-E^d^ as major histocompatibility on the tumor vascular endothelium. Enhanced coagulation was observed in vitro and in vivo. Furthermore, it is indicated that 38% of murine models that received B21-2/10HlO-tTF coagulant had whole tumor regressions. This event endured four months or more [269]. Using cell adhesion molecules for targeting tumor vessels has been investigated by several studies. Vascular cell adhesion molecule-1 (VCAM-1) is expressed by endothelium in Hodgkin's disease and different cancers like small-cell lung carcinoma. It is not present in normal endothelium of mice, except heart and lungs. It has been reported that the treatment of murine models with different types of cancers by anti-VCAM-1 led to thrombosis and tumor necrosis in those neoplasm regions. In contrast to the former study, complete tumor regressions were not observed in Hodgkin's tumors in mice, and in all murine models, tumors regrew from persisting tumor cells in VCAM-1-negative areas. Anti-VCAM-1-tTF was localized in the heart and kidney vessels but did not induce thrombosis after binding to the endothelium. The VCAM-1-expressing normal vessels in those regions were not thrombosed because they lack PS on their luminal surface [270, 271].

Fibronectin is another adhesion molecule that accumulates in neovascular structures in tissues undergoing angiogenesis, such as aggressive tumors. ChTV.1-tTF targets fibronectin on vessels, leading to thrombosis in small and medium vessels [272]. The extra domain B of fibronectin (ED-B) is identical between mice and humans; therefore, Nilsson and colleagues showed that targeting the ED-B domain by an antibody fragment combined with the tTF led to selectively targeting tumor blood vessels in vivo. They designed ScFv(L19)-tTF, which displays a highly accumulated neovasculature a few hours after injection and led to whole tumor cells eradication in 30% of the mice treated in the absence of noticeable side effects at the highest doses of administration in three various kinds of tumors [273, 296].

Using monoclonal antibodies against nuclear-related antigens has been investigated. Tumors may contain high proportions of degenerating cells with areas of frank necrosis. Epstein and colleagues have hypothesized that tumors containing dead or degenerating malignant cells may be readily distinguished from normal tissues. They designed two monoclonal antibodies, TNT-1 and TNT-2, with specificity against nuclear-related antigens [297]. In the subsequent study, TNT-3 and chimeric TNT-3 (chTNT-3) were designed, illustrating threefold higher tumor uptake than TNT-1 [298]. It was observed that vessel thrombosis was caused by chTNT.3-tTF occurred mainly in larger ones. Blocking of tumor blood supply, necrosis of tumor cells, and then significant tumor growth inhibition was observed in chTNT-3-tTF-treated tumor-bearing mice without any obvious dose–response effect [272].

A monoclonal antibody attached to factor VIIa was designed by Rippmann and colleagues. They produced a TFOS4, a single-chain humanized antibody bound to fibroblast activation protein (FAP). Activated fibroblasts in the tumor stroma especially and extensively express this marker. Selectively targeting tumor cells by TFOS4 could induce plasma coagulation, whereas intravenously injection into normal murine models illustrated no systemic coagulation or unfavorable effects [275].

Peptide-mediated targeted tTF may have more merits over antibodies, especially larger ones. Low tumor penetration and potential immunogenicity are two significant side effects of larger antibodies. Further, reticulohistiocytic systems (RHS), such as in bone marrow, liver, and spleen, could uptake antibodies that might bring about potentially harmful induction of blood clotting in these organs [284, 299, 300].

Pasqualini & Ruoslahti showed that peptides could localize phages, particularly to the brain and kidney, and showed up to 13-fold selectivity for these organs for the first time [301]. One appealing synthetic peptide that can be recognized by cell surface integrins is Arg-Gly-Asp tripeptide (RGD). Peptides containing RGD motifs originated from matrix proteins like fibronectin, vitronectin, etc. [302]. It has been reported in different studies that cyclic RGD could bind to α_5_β_1_, α_v_β_3_, and α_v_β_5_ integrins, and cyclic phage peptides have higher affinities for the integrin than linear peptides [303–305]. αvβ3 and αvβ5 integrins were present in tumor vessels of some kinds of cancers and targeted by RGD-tTF. Thrombosis caused by RGD-tTF mainly occurred in capillaries and small vessels, causing insignificant harm to the tumor in vivo, and did not have inhibitory effects on tumor growth. In a subsequent study, Kessler et al. developed tTF-RGD as a fusion protein that consists of tTF and a RGD peptide bound to the c-terminal domain of tTF. They reported that administration of tTF-RGD brought about thrombotic occlusion of tumor vessels and hindered tumor growth in mice in the absence of any unfavorable effects. These apparent antitumor activities of tTF-RGD are opposed to the study of Hu and colleagues. However, tumor regrowth was reported after termination of therapy [272, 276].

VEGFR-2 and neuropilin-1 are highly expressed by endothelial and tumor cells [278, 306]. VEGF and semaphorins could bind to the neuropilin-1 (Npn-1) as their receptor. Semaphorins are a large family that primarily act as neuronal mediators [307]. Besides, Npn-1 seems to have a critical role in tumor angiogenesis and the conversion of benign stromal tissues to malignant ones [308–310].

As part of the heparin-binding domain of VEGF, HBDt has been shown to engage in a trimolecular complex of chondroitin C sulfate proteoglycan, neuropilin-1, and VEGF receptor-2. Administration of HBDt that fused to tTF brought about thrombosis in tumor vessels, decreased tumor size, and almost eradicated them [278]. Subsequently, functions of tTF-EG3287 and SP5.2-tTF were investigated, respectively targeting neuropilin-1 and VEGFR-1. tTF-EG3287 blocked tumor-supplying vessels that reduced tumor size, and SP5.2-tTF showed favorable antitumor activity during treatment [280, 281].

It has been revealed that shorter TF molecules (TF _1–214_) showed stronger pro-coagulatory activity than the complete extracellular domain (TF _1–219_) [311]. For more investigation, Brand et al. have designed tTF-TAA, tTF-LTL, and tTF-NGR with various sizes of the tTF-moiety. These fusion proteins represent ligands of NG2. NG2 (also called nerve/glial antigen 2) is a transmembrane proteoglycan expressed in numerous different cell types, specifically tumor vessel walls or pericytes on the abluminal surface of endothelial cells. In contrast to normal vessels, tumor vessels show a permeable endothelium. So, it becomes a probable target for the vascular delivery of antitumor agents and strategies. Only tTF-LTL, which consists of the selected length of the TF sequence, was observed with varied pro-coagulatory activity, but in contrast to the former study, this variation was not significant. Although they reported vascular obstruction and decreased tumor blood supply by utilizing tTF-TAA, it showed a small therapeutic window [284, 312].

In addition to NG2, it was revealed that NGR-containing peptides couple to aminopeptidase N (APN; so-called CD13), α_v_β_3_, and α_5_β_1_ [304, 313]. The tTF-NGR suppressed tumor development in mice via thrombotic blockage of blood flow in tumor vessels, with no major adverse effects in other organs at therapeutic doses but not in high doses. Also, it was demonstrated suppression of tumor perfusion in the clinical first-in-man administration of this molecule at low doses [284, 285].

As shown previously, ChTV.1-tTF and ScFv (L19)-tTF represent fibronectin ligands, accumulating in tumors and inducing intratumoral thrombosis [272, 273]. Then Shi and colleagues produced a new fusion protein, tTF-CREKA [286]. Cys-Arg-Glu-Lys-Ala (CREKA), a phage display-identified tumor-homing pentapeptide, identifies microthrombus-associated fibrin-fibronectin complexes [289, 292]. They observed that tTF-CREKA reduced tumor development with more efficacy and at a lower dosage than some other fusion proteins. Therefore, it seems to be a safe cancer therapeutic approach. After systemic treatment, this suppression occurred due to intratumoral thrombosis induction [286].

Combination therapy of vascular targeting agents, besides other treatment approaches, has been investigated. Liu and colleagues targeted PMSA concurrent with the administration of doxorubicin [287]. Prostate epithelial cells can be identified by the PSMA protein. However, normal prostate epithelial cells generate a cytosolic version of PSMA that is highly expressed by carcinoma of the prostate, nearly a 100-fold increase [314, 315]. Targeting PMSA alone resulted in strong and highly selective tumor microvascular thrombosis. For combination therapy, 2 mg/kg of liposomal doxorubicin (Doxil) was administered intravenously. This Combination therapy was far more effective, greatly increased tumor eradication, and dramatically extended the period during which the patient remained tumor-free. Considering that doxorubicin causes endothelial cells to undergo apoptosis, it may boost local thrombotic activity by increasing tumor cell antigen exposure to plasma proteins. However, this investigation showed that doxorubicin treatment alone had no noticeable impact on tumor growth or survival [287, 316].

Some new strategies have been investigated to enhance the therapeutic efficacy of vascular targeting. Using a two-step coagulation approach by utilizing the streptavidin–biotin system was explored recently. XU and colleagues carried out a study using a streptavidin-conjugated anti-neuropilin-1 monoclonal antibody (mAb-SA) and biotinylated tTF (tTF-B) [288]. As previously demonstrated, neuropilin-1 is the proper target on the surface of tumor vascular endothelium [278, 306]. First, mAb-SA was diffused into the tumor region, followed by the administration of tTF-B, which effectively interacted with mAb-SA to cause tumoral thrombosis. In vitro and in vivo investigations have shown that the mAb-SA:tTF-B approach hindered tumor development and elevated tumor regression by targeting tumor blood vessels selectively and generating total vascular infarction [288].

One more practical approach is utilizing a nanoparticle delivery system mimicking platelets. These particles increase their own homing and amplify their accumulation like platelets. Simberg et al. coupled CREKA as a tumor-homing peptide to nanoparticles and liposomes. They observed a 20% obstruction rate, but this vessel occlusion degree is insufficient to reduce the tumor growth rate. They suggest that optimizing the formulas and methods could change the degree of vessel occlusion [289, 292].

A fusion protein (tTF-pHLIP) consists of tTF fused to a peptide with a low pH-induced transmembrane structure is another approach for delivering tTF. pHLIP is generally water-soluble at physiological pH, but at the slightly acidic pH of tumor vasculature, it forms α-helix capable of insertion into a lipid bilayer. In preclinical studies, systemic injection of tTF-pHLIP selectively triggered thrombotic obstruction of tumor vessels, therefore decreasing tumor perfusion and inhibiting tumor development without major adverse consequences [264].

Since some peptides and antibodies could mediate selective localization on tumor vessels, some studies set out to apply them to facilitate cancer detection and deliver tumor inhibitor agents, such as anti-cancer drugs, nanoparticles, and cytokines, into tumor tissues [291–294].

For example, RGD and NGR sequences were coupled to doxorubicin as chemotherapeutic agents. This experiment led to favorable antitumor activity during treatment and prolongation of survival [291]. Moreover, intravenously administered liposomes coupled to CREKA and delivered ticagrelor as a platelet inhibitor led to inhibit tumor metastasis without overt side effects [292]. Epstein and colleagues generated a monoclonal antibody (TV-1) targeting a basement membrane antigen found in all tissues but accessible only in tumor vessels. Administration of TV-1 which was bound to IL-2, enhanced the uptake of radiolabeled tumor-specific monoclonal antibodies [293].

One of the outstanding techniques for high-resolution visualization of the anatomic structure of soft tissues like tumors is magnetic resonance imaging (MRI). Early detection and accurate diagnostic imaging of high-risk breast cancer are crucial in selecting suitable and effective strategies and interventional therapeutic agents. Therefore, Zhou and colleagues bound a pentapeptide CREKA to an MRI contrast agent and developed CREKA-Tris (Gd-DOTA)3 for molecular visualization with contrast-enhanced MRI. It was observed that CREKA-Tris (Gd-DOTA)3 could effectively visualize metastases containing micrometastases in aggressive breast cancer [294].

### Conclusion and future prospectives

Cancer progression is significantly influenced by TF, which has been identified as a potential therapeutic target. Tissue factor is overexpressed in a wide range of cancers, and its expression is correlated with poor patient outcomes. Tumor growth, angiogenesis, and metastasis can be inhibited by targeting TF, resulting in improved patient survival. Agents such as monoclonal antibodies, small molecules, CAR T-cells, and RNA interference have been developed to target TF.

One promising area of research in the field of TF-targeted therapy is the use of CAR T-cells that target TF. In preclinical models of cancer, CAR T-cells targeting TF inhibit tumor growth and extend survival. As well as exhibiting potent cytotoxicity against TF-expressing tumor cells, these CAR T-cells have also been shown to infiltrate solid tumors and promote immune cell infiltration. Even though CAR T-cell therapy has shown remarkable success in treating certain cancers, it also faces significant challenges. Potential toxicity is one of the major limitations, particularly cytokine release syndrome and neurotoxicity. Moreover, CAR T-cell therapy is expensive and complex, making it inaccessible to many patients. The development of CAR T-cells targeting TF remains a promising approach for treating cancer. This approach has the potential to revolutionize cancer therapy and improve patient outcomes, but further research is needed to optimize CAR T-cell design.

In addition, re-targeted therapies have opened up new avenues for treating cancer. Through re-targeted therapies, therapeutic agents are delivered directly to cancer cells. In addition to reduced toxicity and improved pharmacokinetics, this approach has the advantage of targeting multiple antigens simultaneously. In preclinical models of cancer, re-targeted therapies have shown promising results when TF-targeting antibodies are coupled with therapeutic agents. It is, however, still difficult to translate TF-targeted and re-targeted therapies into clinical practice, which remains a significant challenge. The delivery of drugs, toxicity, and resistance are all problems that need to be overcome. The use of these therapies for specific patient populations and the optimization of treatment regimens require further study. Despite the promising results of the new treatment and the FDA approval of Seagen and Genmab's tisotumab vedotin for cervical cancer treatment, it is crucial to acknowledge that further research is necessary to comprehensively grasp TF targeted and re-targeted advantages and limitations [317]. As we learn more about the role of TF in cancer, new therapeutic approaches may emerge that target it more effectively. A TF-targeted and re-targeted therapy may ultimately lead to a cure for cancer with continued research and development.

## Data Availability

Not applicable.
